# Synthesis of Flexible Random Copolymers of Poly(butylene *trans*-1,4-ciclohexanedicarboxylate) Containing Pripol Moiety as Potential Candidates for Vascular Applications: Solid-State Characterization and Preliminary *In Vitro* Biocompatibility and Hemocompatibility

**DOI:** 10.1021/acs.biomac.4c01668

**Published:** 2025-04-30

**Authors:** Edoardo Bondi, Nora Bloise, Michelina Soccio, Giulia Guidotti, Ilenia Motta, Massimo Gazzano, Marco Ruggeri, Lorenzo Fassina, Emilia Genini, Livia Visai, Gianandrea Pasquinelli, Nadia Lotti

**Affiliations:** a Department of Civil, Chemical, Environmental, and Materials Engineering, University of Bologna, Via Terracini 28, Bologna 40131, Italy; b Molecular Medicine Department (DMM), Centre for Health Technologies (CHT), Unità di Ricerca (UdR) INSTM, University of Pavia, Pavia 27100, Italy; c UOR6 Nanotechnology Laboratory, Department of Prevention and Rehabilitation in Occupational Medicine and Specialty Medicine, Istituti Clinici Scientifici Maugeri IRCCS, Via Maugeri 4, Pavia 27100, Italy; d Interuniversity Center for the Promotion of the 3Rs Principles in Teaching and Research (Centro 3R), Operative Unit (OU) of University of Pavia, Pavia 27100, Italy; e Department of Medical and Surgical Sciences (DIMEC), University of Bologna, Via Massarenti 9, Bologna 40138, Italy; f Institute for Organic Synthesis and Photoreactivity, ISOF-CNR, Via Gobetti 101, Bologna 40129, Italy; g Department of Drug Sciences, University of Pavia, Viale Taramelli 12, Pavia 27100, Italy; h Department of Electrical, Computer and Biomedical Engineering, University of Pavia, Via Ferrata 5, Pavia 27100, Italy; i Fondazione IRCCS Policlinico San Matteo, Pavia 27100, Italy; j Pathology Unit, IRCCS Azienda Ospedaliero-Universitaria di Bologna, Bologna 40138, Italy

## Abstract

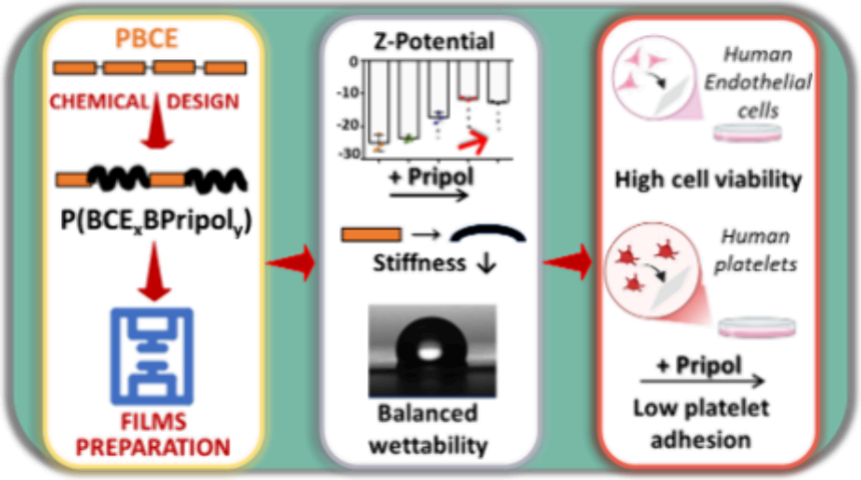

In order to envisage new solutions for complications associated with cardiovascular diseases, including the occlusion of small vessels, a family of random copolymers of poly(butylene *trans*-1,4-ciclohexanedicarboxylate) (PBCE), containing Pripol moiety, namely, poly(butylene *trans*-1,4-ciclohexaendicarboxylate/Pripol), were successfully synthesized. The copolymers display reduced crystallinity and stiffness compared with PBCE, exhibiting elastic modulus values that are comparable to those of materials previously investigated for similar applications. The stability of the materials under physiological conditions was demonstrated over an extended time. Cytotoxicity was confirmed by a direct contact assay with human umbilical vein endothelial cells (HUVECs), and blood compatibility was established by the absence of any change in the values of activated prothrombin time and activated partial thromboplastin time, in addition to the low adhesion of blood components. The results demonstrated that the ad hoc design is pivotal in regulating solid state and functional properties, thereby facilitating the development of innovative materials for vascular tissue engineering.

## Introduction

Cardiovascular diseases (CVDs), called also the “silent killer” of our century, are the leading cause of death globally and one of the major contributors to disability according to The World Health Organization. CVDs are globally responsible for more than 17 million deaths/year, which correspond to about one-third of all deaths.^1,2^ These alarming numbers are expected to even rise to over 23 million by 2030. In the European Union, CVDs cause about 1.8 million deaths every year, which corresponds to 5000 deaths per day, and represent a costly burden for society,^3^ with an overall cost of 210 billion Euros per year.^4^

Despite huge advances in cardiovascular medicine, CVDs are often the result of behavioral risk factors, such as unhealthy diet, physical inactivity, and harmful use of tobacco and alcohol, and can lead to serious complications like stenosis or occlusion of blood vessels, coronary artery and cerebrovascular diseases, deep vein thrombosis, and atherosclerosis.^5^ Among these, peripheral artery disease (PAD) is an occlusive disease of the lower extremities and, in particular, of tibiopedal vessels located below the knee, being the primary cause of critical limb ischemia (CLI), a severe blockage in the arteries of the lower extremities. If not properly or promptly treated, patients with CLI have a poor prognosis: almost one-third of people have an amputation and, worse yet, about one in four dies.^6,7^ The most common procedure to restore a proper blood flow involves revascularization with endovascular procedures, such as angioplasty or atherectomy, but when these are not applicable, that is, in case of long lesions, calcified arteries and chronic total occlusions, open surgery remains the best choice.^8^ In this regard, autografts are the reference procedure because of a limited risk of immune reaction and matching mechanical properties. However, due to donor sites morbidity and limited availability, the search for a more effective alternative is urgently required.^5^ To this aim, artificial grafts have been implemented and successfully used into large-diameter bypass (>6 mm). Conversely, small-diameter synthetic grafts are an issue still unresolved, being often associated with complications like infections, thrombosis, intimal hyperplasia, calcification, and aneurysm formation, with a high failure rate.^9−11^ The development of innovative tissue engineering and regenerative medicine techniques can overcome these limitations, allowing for the fabrication of grafts that can interact with the surrounding environment and remodel the host vessel.

To be suitable for implantation in human body, a biomaterial should mimic the natural surrounding environment as closely as possible, in order to ensure mechanical, functional, and biological compatibility.^12^ In particular, for vascular tissue engineering, also hemocompatibility, nonimmunogenicity, and bioactivity must be considered.^13^

Polymers and, in particular, aliphatic polyesters like polylactic acid (PLA), polyglycolic acid (PGA), poly(ε-caprolactone) (PCL), and their copolymers, have been widely investigated and used for biomedical purposes.^14^ Some of them^15−17^ have been also tested as small-diameter vascular grafts in large animals and humans.^18^ Inside this wide family, poly(butylene *trans*-1,4-cyclohexanedicarboxylate) (PBCE) has attracted increasing interest in the past few years thanks to its proven biocompatibility, good thermal resistance, and easy processability. On the contrary, although its high crystallinity and mechanical rigidity are suitable, for example, for the restoring of neuronal musculoskeletal tissues,^19−23^ its applications in soft and vascular tissue engineering are still limited. As is well-known, a way to improve the unsatisfactory properties of a polymer, maintaining at the same time the already good ones for a selected application, is copolymerization. Many studies have been already published in the literature about blends^24,25^ and copolymeric systems containing cyclohexane ring,^26−30^ for biomedical or packaging applications,^31−34^ in order to investigate the effect of *cis*/*trans* ratio^35 −37^ or to tune the crystallization capability^38−42^ of the final materials. However, to the best of our knowledge, PBCE copolymers containing the Pripol 1009 subunit have not yet been studied to date. Pripol 1009 is a commercial fatty diacid obtained from renewable sources, currently used for the production of adhesives,^43^ elastomers, and self-healing materials for cutting-edge applications.^44−47^ It contains an aliphatic six-carbon ring connected to two −COOH groups through PE-like segments and presents also two long alkyl chains, which are known to reduce the crystallization capability of the final material. Thus, the peculiar chemical structure of Pripol 1009 can enhance the flexibility of the new copolymers. Moreover, this subunit allows reducing the −COOR– density per chain unit, limiting thermal degradation caused by cleavage of ester linkages and thus increasing the stability.

The family of copolymers, namely, poly(butylene *trans*-1,4-ciclohexaendicarboxylate/Pripol), P(BCE_*x*_BPripol_*y*_), where *x* and *y* represent the relative molar amount of the two counits, was synthesized starting from four different molar compositions between the diacid subunits and then characterized from the chemical, structural, thermal, and mechanical point of view. The degradation rate under both physiological and accelerated conditions was also evaluated to check the suitability of these materials in the human body for long-term applications. Since the ideal vascular grafts should be biocompatible and hemocompatible,^13,48^ after a preliminary *in vitro* cytotoxicity evaluation was carried out by cell viability studies, the effects on surface *Z*-potential, the surface texture features and hemocompatibility of the compression-molded films of the synthesized materials were examined. Specifically, the surfaces were investigated for different aspects of hemocompatibility, including the effect on the coagulation process, the human fibrinogen binding, and the platelet adhesion.

## Materials and Methods

### Materials

*Trans*-1,4-cyclohexanedicarboxylic acid (CHDA, Fluorochem), 1,4-butanediol (BD, Sigma-Aldrich), titanium tetrabutoxide (TBT, Sigma-Aldrich), Pripol 1009 (kindly supplied by Croda Italia S.p.A.), and 3-(4,5-dimethylthiazole-2-yl)-2,5-diphenyl tetrazolium bromide (MTT, Sigma-Aldrich) were all used as purchased.

### Synthesis

**Scheme 1:**
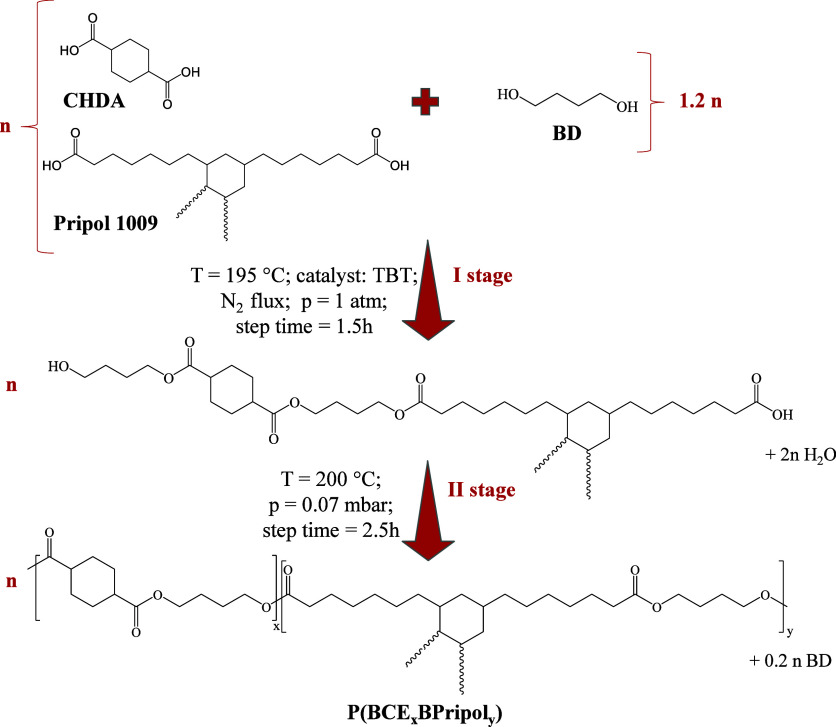
Schematic Representation of the Synthetic Procedure

The copolymers poly(butylene *trans*-1,4-cyclohexanedicarboxylate/Pripol), P(BCE_*x*_BPripol_*y*_), (*x* and *y* are the relative molar amounts of the two diacid counterparts) were obtained by melt polycondensation of CHDA and Pripol 1009, in the proper molar ratios, with BD. This last was added with an excess of 20 mol % with respect to the diacid counterpart, in the presence of the catalyst TBT (400 ppm/g of polymer). The reagents and the catalyst were all charged in a continuously stirred glass reactor, which was put in a thermostatic bath. The synthesis consists of two steps, as shown in Scheme 1. Briefly, in the first step, carried out at 195 °C for 90 min in an inert atmosphere (N_2_), esterification reactions take place, with the formation of oligomers and water molecules, these last collected in a trap connected to the reactor. In the second step, started when the 90% of the theoretical water has been collected in the trap, the temperature was increased to 200 °C and the pressure was slowly decreased to 0.070 mbar. In this stage, transesterification reactions take place, with the progressive increase of polymer’s molecular weight. The low pressure permits the removal of the volatile byproducts as well as the glycolic excess. The synthesis was stopped after about 2.5 additional hours, when a high and constant value of torque was reached. The homopolymer PBCE was also synthesized, for the sake of comparison, under the same conditions.

Prior to processing, the so-obtained materials were purified by dissolution in chloroform under stirring and further precipitation in a large molar excess of methanol.

### Molecular Characterization

The chemical structure and composition, as well as the randomness degree (*b*) and block length (*L*), were determined by ^1^H NMR and ^13^C NMR spectroscopy, respectively. The samples were dissolved (with a concentration of 10 and 40 mg/mL for ^1^H NMR and ^13^C NMR, respectively) in deuterated chloroform with tetramethylsilane (TMS, 0.03 vol%) as an internal standard. The measurements were carried out at 25 °C by employing a Varian INOVA 400 MHz instrument.

Gel permeation chromatography (GPC) was used to measure the molecular weight (*M*_n_) and polydispersity index (*Đ*) of the synthesized polymers. The analyses were carried out at 25 °C using a LabFlow 2000 (Wyatt Technology) instrument and equipped with a Phenomex (300 × 7.8 mm 5 μm) column and a refractive index detector Knauer K-2301. The system was eluted with chloroform at a rate of 0.3 mL/min, and polystyrene standards (2000–100,000 g/mol) were used to obtain a calibration curve.

### Film Preparation

All of the purified samples were compression molded with a Carver C12 lab press. The polymers were placed between two Teflon plates, heated to a temperature 40 °C higher than their melting temperature, and let them melt for a couple of minutes. Afterward, a pressure of 5 ton/m^2^ was applied and kept for 3 min; last, the films were ballistically cooled to room temperature under pressure.

Prior to further characterization, films were stored at room temperature for 2 weeks in order to let them reach crystallization equilibrium.

### Thermal Analysis

Thermal stability was evaluated through thermogravimetric analysis (TGA), by means of a PerkinElmer TGA 4000 instrument. The measurements were carried out under a nitrogen atmosphere (40 mL/min) by heating weighed samples of about 10 mg from 40 to 800 °C, at a rate of 10 °C/min. The temperature of maximum degradation rate, *T*_max_, was calculated from the TGA curve derivatives as the minimum of the peak, while the temperature of initial degradation, *T*_id_, represents the temperature at which degradation starts.

To define the characteristic thermal transitions of the polymers, differential scanning calorimetry (DSC) was carried out using a PerkinElmer DSC6 instrument equipped with an intracooler able to reach −70 °C. Samples (about 8 mg each) were subjected to the following thermal treatment under a nitrogen atmosphere (20 mL/min):

- heating step from −70 °C to *T*_m_ + 20 °C at 20 °C/min (I scan);
- isothermal step of 3 min;
- cooling step to −70 °C at 100 °C/min;
- isothermal step of 17 min;
- heating step from −70 °C to *T*_m_ + 20 °C at 20 °C/min (II scan).

Glass-transition temperature (*T*_g_) was taken at half-height of the glass-to-rubber transition step, while the corresponding heat associated (Δ*C*_p_) was calculated from the step height; melting temperature (*T*_m_) was taken as the maximum of the endothermic melting peak, while the associated enthalpy (Δ*H*_m_) was calculated from the area under the peak.

### Wide Angle X-ray Scattering (WAXS)

Wide angle X-ray scattering (WAXS) analysis was carried out at room temperature with a PANalytical X’Pert PRO diffractometer equipped with an XCelerator detector. The Cu anode was used as the X-ray source (λ_1_ = 0.15406 nm, λ_2_ = 0.15443 nm). The degree of crystallinity (χ_c_) was evaluated as the ratio between the areas of crystalline peaks and the total diffraction area under the scattering curve, using a HighScore software 4.9 release (PANalytical).

### Water Contact Angle (WCA) Measurements

Static water contact angle (WCA) measurements were performed on flat film surfaces using a Krüss DSA30S instrument equipped with Drop Shape Analysis software. Each sample was washed previously using a 70% v/v ethanol solution and then dried off overnight at room temperature. The profile images of deionized water drops (4 μL) were acquired after 1 s from the deposition, and WCA values were measured by means of a software. At least 10 measurements were performed for each film, and the WCA was calculated as the average ± standard deviation.

### Mechanical Characterization

Tensile and cyclic testing were performed using an Instron 5966 machine equipped with rubber grips and a 10 kN load cell controlled by a computer. For both tests, rectangular stripes (5 mm × 50 mm, a gauge length of 20 mm) were employed, and a crosshead speed of 10 mm/min was adopted. Load–displacement values were converted to stress–strain curves.

As to the tensile testing, stress at break (σ_B_) and elongation at break (ε_B_) were determined from the end point of the relative stress–strain curve, while the tensile elastic modulus (*E*) was calculated from the slope of the initial linear part. For the cyclic tests, specimens were strained until they reached a deformation well below their ε_B_ (20 and 50%), and then, 20 cycles at a rate of 10 mm/min were carried out for each elongation. The hysteresis energy (*U*_hys_) was calculated from the area between the loading and unloading curve and the percentage recovery (*r* %) was calculated as
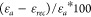
where ε_a_ and ε_rec_ are the applied and the recovery elongation, respectively. For both tests, at least six specimens were analyzed, and the results have been provided as the average value ± standard deviation.

### Hydrolytic Degradation Tests

Hydrolytic degradation tests were carried out in phosphate-buffered saline solution (PBS, 137 mM NaCl, 2.7 mM KCl, 4.3 mM Na_2_HPO_4_, 1.4 mM KH_2_PO_4_, pH 7.4) at 70 °C (accelerated condition) for 60 days according to ISO 10993-13^49^ and at 37 °C (physiological conditions) for 6 months. For each material under examination, rectangular samples (0.5 mm × 2 mm) were used. Each piece was weighted, then immersed in 2.5 mL of PBS, and incubated at 70 °C in an MPM Instruments M-80 VF oven (accelerated conditions) and at 37 °C in a SW22 Julabo shaking water bath (physiological conditions). Blank samples were also incubated under the same temperatures, but without PBS, for the sake of comparison. The buffer solution was changed periodically to keep the pH constant for the entire time scale of the degradation experiment. Gravimetric and molecular weight loss measurements as well as DSC analysis of partially degraded samples were performed. More in detail, after the fixed times, duplicate specimens and the relative blank were removed and dried off to a constant weight. The percentage weight loss was calculated as
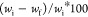
where *w*_f_ and *w*_i_ are the final and the initial weight of the sample, respectively. At the same time points, the percentage molecular weight loss was also calculated as
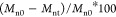
where *M*_n0_ and *M*_nt_ are the initial molecular weight and the molecular weight at time *t*, respectively.

The kinetic constant was obtained according to Pitt et al.:^50^
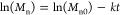
by plotting the natural logarithm of *M*_n_ as a function of the degradation time (*t*).

### Surface Zeta Potential

The zeta potential (ζ) of each film at physiological pH was determined from the measurement of the streaming potential as previously reported.^51^ The apparent zeta potential (ζ) of each sample was determined using SurPASS 3 (Anton Paar GmbH, Austria). The samples (75 mm^2^) were placed between two filter disks in the sample holder of the cylindrical cell. Potassium chloride aqueous solution (0.01 M) was used as the streaming solvent, and ζ was measured at pH 7.4.

### Texture Features

The surface of the biomaterials was evaluated via a Computer Vision approach, in particular by the so-called Haralick’s texture features.^52^ Originally, the Haralick’s features were 14 but, and in the present study, new formulas given by Löfstedt et al.^53^ were employed, which defined 7 new features and make all the 21 Haralick’s features independent of image quantization and, thus, more suitable for Computer Vision.

In detail, by a custom-made script written in Matlab Programming Language (Release R2022b, The MathWorks, Inc., Natick, MA, USA), from each SEM image, the 21 Haralick’s texture features were extracted. A multivariate repeated measures statistics based on the Tukey’s Honest Significant Difference procedure was also performed. In particular, the predicting variables were the 21 Haralick’s texture features, while the predicted variable was the biomaterial type. Each one-to-one comparison between biomaterials was expressed in terms of *p*-value (selecting a significance level of 0.05).

### In Vitro Biological Evaluation

#### Cytotoxicity Tests

##### Cell Culture

Human umbilical vein endothelial cells (HUVECs) were cultured routinely in a Dulbecco’s Modified Eagle Medium (DMEM), supplemented with 10% fetal bovine serum (FBS) in incubator at 37 °C and 5% of CO_2_. Before the experiment, the polymer films (1 × 1 cm) were sterilized in aqueous solutions containing increasing concentrations of ethanol (70 and 90 vol%) for 30 min each and then washed twice with PBS and cell culture medium for 10 min.

##### MTT Assay

HUVECs were seeded in a 24-well plate with a number of 5000/cm^2^ for each well, and after 5 h of incubation, the sterilized polymers were put inside the wells. The plate was kept in an incubator at 37 °C and 5% of CO_2_. The viability of HUVECs was evaluated by means of a 3-[4,5-dimethylthiazol-2-yl]-2,5 diphenyl tetrazolium bromide (MTT) assay, which was carried out after 1 and 3 days of incubation. More in detail, at the desired time points, both polymer and cellular medium were removed from the wells. Then, 450 μL of DMEM and 50 μL of MTT solution were added to cells. After 2.5 h of incubation, 500 μL of lysis buffer (0.1 g/mL sodium dodecyl sulfate, 41.67 μL/50 mL HCl 0.01 M) was added, and the day after, aliquots of 200 μL of solution were sampled. The absorbance values were measured at 570 nm by means of a microplate reader.

#### Hemocompatibility Assays

##### Clotting Time

Sterilized tested materials were cut into squares of 1 × 0.5 cm size and placed in a test tube followed by addition of 300 μL of human Platelet-Poor Plasma (PPP) in order to measure prothrombin time (PT) and activated partial thromboplastin time (APTT).^54,55^ After 60 min of incubation at 37 °C, the film was removed from a test tube and both PT and APTT were measured using an automatic analyzer BCS 5100XP (Siemens) according to the manufacturer’s instructions. Test tubes containing only 300 μL of PPP without any material present were used as controls.

##### Platelet Adhesion

Human platelet-rich plasma (hPRP) was obtained from Fondazione IRCCS Policlinico San Matteo, Pavia (Italy). hPRP was isolated according to “Decreto Ministero della Salute 2 November 2015 n.69, Disposizioni relative ai requisiti di qualità e sicurezza del sangue e degli emocomponenti” and “Accordo Stato-Regioni n.225/CSR 13 December 2018, Schema-tipo di convenzione per la cessione del sangue e dei suoi prodotti per uso di laboratorio e per la produzione di dispositivi medico-diagnostici in vitro”. hPRP (containing 2 × 10^8^ platelets/mL) in 10 mM EDTA solution (Sigma-Aldrich, St. Louis, MO, USA) was added to each well containing the previously sterilized sample and incubated at 37 °C for 60 min. After that, the samples were washed with PBS 1× to remove no adherent platelets and prepared for quantitative lactate dehydrogenase (LDH) activity and qualitative scanning electron microscopy (SEM) examination of adherent platelets. For the quantification of platelet adherence on each sample, the LDH assay (Sigma-Aldrich) was employed. After the incubation, adherent platelets were lysed by adding 1% Triton buffer (Triton X-100, Sigma) to each well and LDH release was quantified according to the manufacturer’s instructions. A titration curve with known concentration of platelets/mL was used to plot the obtained absorbance. Platelet’s adhesion was expressed as percentage related to initial platelets incubated on each sample set as 100%. For the qualitative observation by SEM, after 60 min incubation, samples were fixed with 2.5% (v/v) glutaraldehyde solution in 0.1 M Na-cacodylate buffer (pH = 7.2) for 60 min at 4 °C, washed with Na-cacodylate buffer, dehydrated at room temperature in an ethanol gradient series up to 100%, and then lyophilized for 4 h for complete dehydration. Samples were then sputter coated with gold and observed with a Zeiss EVO-MA10 SEM (Carl Zeiss, Oberkochen, Germany) at 3000 and 10000× magnification. Platelets seeded on plastic cell-culture coverslip disks (Thermanox Plastic, Nalge Nunc International, Rochester, NY) were used as the control.

#### Protein Quantification

##### Human Plasma Protein Absorption

To quantify the plasma protein adsorption onto each type of sample, all samples were cut into 0.32 cm^2^ pieces of circular shape and incubated with 100 μL of plasma from healthy adult donors for 1 h at 37 °C. After three washes with PBS 1× to remove unbound proteins, the total adsorbed protein content was assessed by the bicinchoninic acid (BCA) assay (EuroClone S.p.A) according to the manufacturer protocol. The absorbance (595 nm) was measured with a microtiter plate reader, the CLARIOstar Plus Multimode Microplate Reader (BMG Labtech, Ortenberg, Germany). The measurement was performed in triplicate with a blank control for each specimen. Standard bovine serum albumin (BSA) curve was used to determine the protein concentrations on each sample, and the data were represented as μg of total protein/cm^2^. As positive control, human plasma was absorbed on a high-binding 96-well microtiter plate (indicated as tissue culture plates, CTRL).

##### Human Fibrinogen Absorption

The human fibrinogen (hFbg) adsorption assay was performed using the enzyme-linked immunosorbent assay (ELISA). Briefly, all samples were cut into 0.32 cm^2^ pieces of circular shape and incubated with 100 μL solution of hFbg (10 μg/mL) or 100 μL of plasma from healthy adult donors for 1 h at 37 °C. Then, each sample was washed with PBS 1× containing 0.05% (v/v) Tween 20, blocked by incubating with 300 μL of PBS containing 3% (w/v) BSA for 1 h at 25 °C, and finally incubated for 1 h at 25 °C with 100 μL with horseradish peroxidase (HRP)-conjugated goat antibody anti-hFbg (1:10000 dilution in 1% BSA, ROCKLAND). After the incubation time and the washes, the samples were transferred into clean wells and the reaction was developed with 200 μL/well of OPD tablets (Sigma-Aldrich, St. Louis, MO, USA) and dissolved in double distilled water. The absorbance was measured at 450 nm with 620 nm as the reference wavelength with a CLARIOstar Plus Multimode Microplate Reader. The experiment was performed in triplicate. A calibration curve was made gradually increasing concentrations of purified human fibrinogen protein (from 500 ng to 10 μg/mL) to obtain the amount of hFbg in each sample. As positive control, 100 μL of human plasma or the solution of hFbg (10 μg/mL) was absorbed on a high-binding 96-well microtiter ELISA plate (indicated as CTRL). BSA-coated wells were used as negative controls.

### Statistical Analysis

All experiments were performed in triplicate, unless otherwise indicated. Results are expressed as the mean value ± the standard deviation. The experiments performed with HUVECs were repeated at least three times. To evaluate the effects of the presence of the films on cell viability, compared to the control (cells cultured without film), a two-tailed student’s test was performed. For the multiple analyses of variables, comparisons among the groups were made using one-way analysis of variance (ANOVA), which was followed by Tukey’s post hoc test, electing a significance level of 95% (*p* < 0.05) All statistical calculations were carried out using GraphPad Prism 10.0 (GraphPad Inc., San Diego, CA, USA).

## Results and Discussion

### Molecular Characterization of the New Family of Random Copolymers

Four different copolymers of PBCE, referred to as P(BCE_95_BPripol_5_), P(BCE_85_BPripol_15_), P(BCE_75_BPripol_25_), and P(BCE_65_BPripol_35_), with different molar ratios between the diacid counterparts, were successfully synthesized by polycondensation in the melt, using TBT as a biocompatible catalyst. The final properties have been correlated to the different chemical composition, considering that the introduction, in the main chain, of a bulky counit characterized by long PE-like segments and long aliphatic pendant groups, is responsible for an increased flexibility.

The purified samples were first characterized from a molecular point of view by means of NMR spectroscopy and GPC analysis. The molecular characterization data are collected in Table 1. By observing the *M*_n_ and *Đ* values, obtained by GPC, it can be assessed that all the materials have high and comparable molecular weights, in the range of 41,000–60,000 g/mol, suggesting a good control of the polymerization (Table 1). The values obtained are slightly higher for all the copolymers compared to the one of PBCE. This can be explained cosidering the higher molecular weight of Pripol 1009 compared to CHDA and assuming a quite fix degree of polymerization. The polydispersity indexes observed for the copolymers are all comparable and range from 2.1 to 2.3, while for the parent homopolymer Đ value is of 1.4 (Table 1). This result can be ascribed to the fact that the introduction of a counit in the main chain of a homopolymer may generate a more polydisperse distribution of the molecular weights.

**Table 1:** Molecular Characterization Data of PBCE and P(BCE_*x*_BPripol_*y*_) Copolymers

Sample	Mn	*Đ*	BCE–feed	BCE–effective	*Cis* isomer	*L*_BCE_	*L*_BPripol_	*b*
	g/mol		mol %	mol %	mol %			
PBCE	41,000	1.4			3			
P(BCE_95_BPripol_5_)	50,000	2.2	95	94	3	6.7	1.4	0.90
P(BCE_85_BPripol_15_)	60,000	2.1	85	82	3	5.6	1.4	0.91
P(BCE_75_BPripol_25_)	48,000	2.3	75	71	5	4.3	1.5	0.89
P(BCE_65_BPripol_35_)	49,000	2.3	65	57	7	2.1	1.8	1

**Figure 1:**
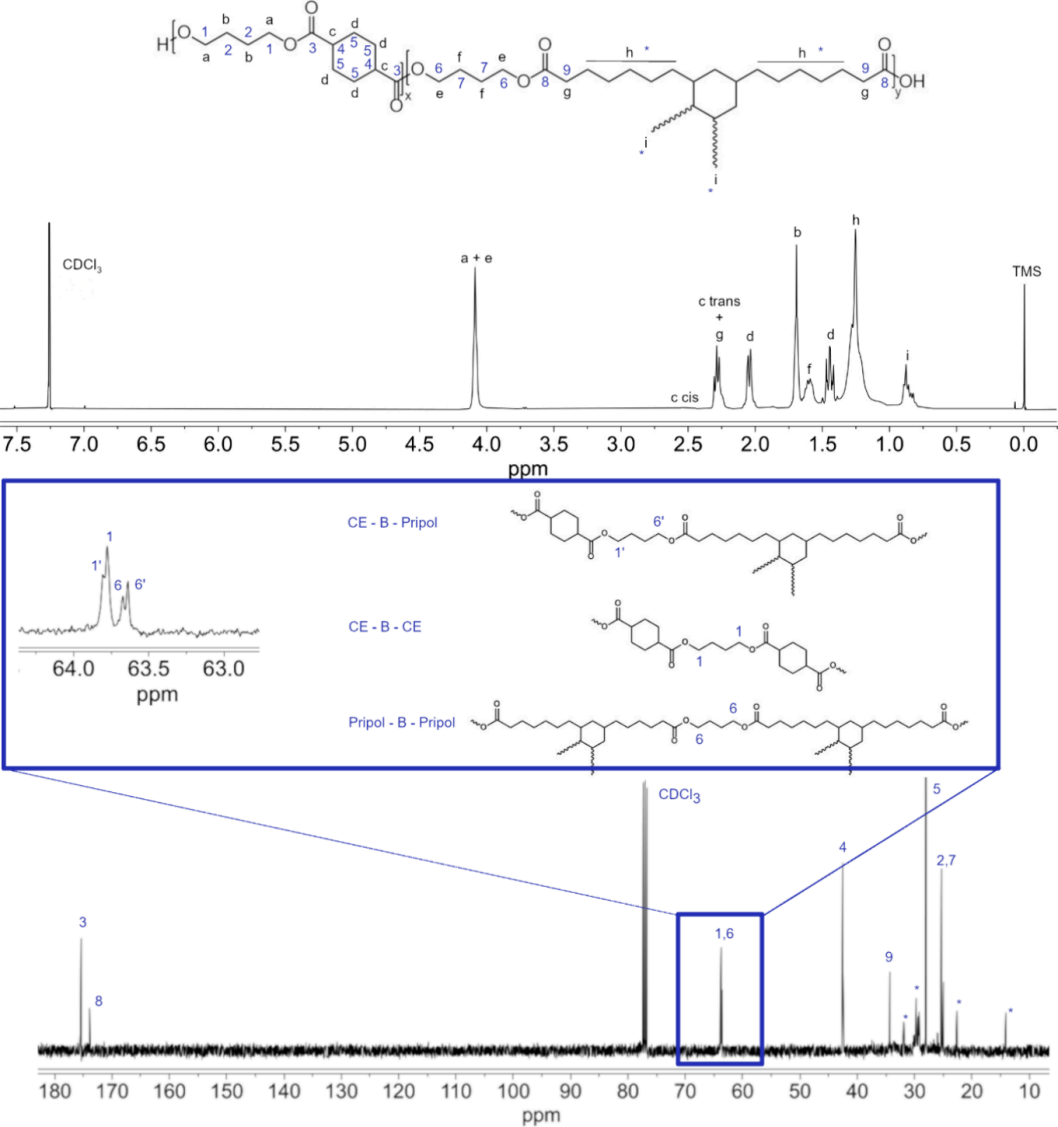
^1^H NMR (top) and ^13^C NMR (bottom) spectra of P(BCE_65_BPripol_35_), with peaks assignment. In the center, an enlargement of the region between 63 and 64 ppm is shown.

By means of ^1^H NMR spectroscopy, it was possible to confirm the chemical composition. As an example, in Figure 1, the ^1^H NMR spectrum of P(BCE_65_BPripol_35_) with the relative peaks’ assignment is reported. In detail, all the peaks of BCE counit are present, being the *a* and *b* signals of methylene protons of the butylene subunit located at δ 4.2 ppm and δ 1.7 ppm, respectively, while the *c*-*trans*, *c*-*cis*, and *d* signals of cyclohexane moiety are located at δ 2.3 ppm, δ 2.5, and δ 2.1 ppm, respectively. As to the peaks of the BPripol unit, the methylene protons *e* and *f* of the glycol subunit are located at δ 4.2 and δ 1.6 ppm, respectively, while the triplet *g* of the protons related to the acid subunit in the α position with respect to the carboxylic group appeared at δ 2.3 ppm. At lower chemical shifts (1.4 ppm < δ < 0.8 ppm), the signals of R pendant groups of Pripol, *h*, and *i* can be seen. The molar ratio between the two diacids was calculated considering the peaks at 2.1 ppm (*d*) for the cyclohexane moiety and 0.9 ppm (*i*) for the Pripol one. In all cases, the effective composition was similar to the feed composition (Table 1), even though the difference between feed and real composition slightly increased with Pripol content may be due to the different reactivities of the two monomers in the melt. Moreover, no additional peaks were found, confirming the good control over the polymerization reaction. At the same time, the *cis* percentage of BCE units in the polymers under study was calculated considering the relative *c* peaks described above, and the results are collected in Table 1. It was found to be comparable for all samples, with values ranging from 3 to 7% and slightly increasing with the amount of counit introduced in the PBCE backbone.

By means of ^13^C NMR spectroscopy, it was also possible to calculate the degree of randomness (*b*) and the length of BCE and BPripol sequences. In the region between 63.5 and 64.5 ppm, the signals related to the −OCH_2_– carbon atoms of butanediol can be found. There are four different signals for the carbons in α-position to the oxygen carbonyl atoms (Figure 1): a peak corresponding to CE-B-CE sequences (1), another corresponding to Pripol-B-Pripol moieties (6) and two peaks corresponding to CE-B-Pripol sequences (1′ and 6′). The degree of randomness (*b*) has been calculated from the relative intensity of these signals according to the following equation:
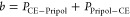
where  and  are the probability of finding a CE subunit next to a Pripol one and the probability of finding a Pripol moiety followed by a CE one, respectively, and “1′”, “1”, “6′”, and “6” are the integrated intensities of the peaks of the CE-B-Pripol, CE-B-CE, Pripol-B-CE, and Pripol-B-Pripol sequences, respectively. Additionally, the length (*L*) of the BCE and BPripol blocks in the copolymeric chain is defined as
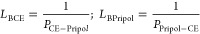


In random copolymers, *b* is equal to 1, whereas is 2 for alternate copolymers and 0 < *b* < 1 for block copolymers. The synthesized copolymers have *b* values very close to 1 (Table 1), suggesting a random distribution of the counits, as expected for copolymers obtained from monomers.

### Physical and Mechanical Characterization of PBCE and Its Random Copolymers

#### Thermal Characterization

**Figure 2:**
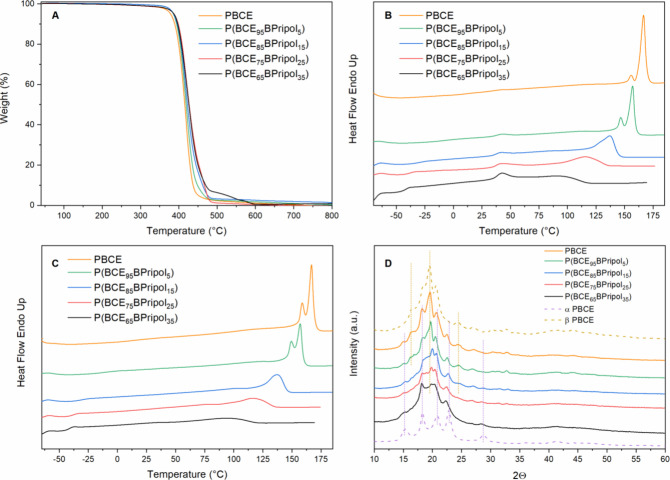
(A) TGA curves of purified powders; (B) I and (C) II DSC scans and (D) WAXS patterns of PBCE and P(BCE_*x*_BPripol_*y*_) compression-molded films.

The thermal stability of the materials under study was studied by thermogravimetric analysis (TGA), conducted under an inert atmosphere. The relative thermograms are shown in Figure 2A, while *T*_id_ and *T*_max_ are reported in Table 2. The PBCE homopolymer presents a high thermal stability (*T*_id_ = 382 °C and *T*_max_ = 405 °C) that becomes even higher by introducing Pripol 1009 in the macromolecular chain. As can be seen from the data reported in Table 2, the higher the amount of Pripol moiety, the more stable the copolymers. This behavior could be attributed to the decrease of −COOR– per unit length as well as to the presence of high thermally stable long PE-like segments in Pripol 1009. It must be noted that these values of thermal stability of the copolymers are among the highest in the class of aliphatic polyesters.^56^ Last, as can be seen from Figure 2A, in all cases, the weight loss reached 100%.

**Table 2:** Thermal (TGA and DSC), Structural (WAXS), and Surface (WCA) Characterization Data of PBCE and P(BCE_*x*_BPripol_*y*_) Copolymeric Films

			DSC											
	TGA		I scan						II scan				WAXS	WCA
	*T*_id_	*T*_max_	*T*_g_	Δ*C*_p_	*T*_1_^a^	ΔH_1_^a^	*T*_2_^a^	ΔH_2_^a^	*T*_g_	Δ*C*_p_	*T*_2_^a^	ΔH_2_^a^	χ_c_	
Sample	°C	°C	°C	J/g·°C	°C	J/g	°C	J/g	°C	J/g·°C	°C	J/g	%	°
PBCE	382	405	10	0.055	42	0.3	166	30	14	0.062	167	26	22	101 ± 5
P(BCE_95_BPripol_5_)	385	410	–9	0.062	42	0.7	157	20	–12	0.080	157	20	19	95 ± 3
P(BCE_85_BPripol_15_)	392	419	–29	0.160	41	1	137	19	–30	0.142	138	16	13	91 ± 2
P(BCE_75_BPripol_25_)	388	414	–38	0.197	41	1	116	12	–37	0.269	119	8	11	93 ± 4
P(BCE_65_BPripol_35_)	395	422	–42	0.296	45	3	98	4	–41	0.286	101	4	9	94 ± 3

a *T*_1_: temperature of isotropization; *T*_2_: temperature of melting; ΔH_1_: enthalpy of isotropization; ΔH_2_: enthalpy of melting.

The materials under study, in the form of purified powders, have been also analyzed by differential scanning calorimetry (DSC). The I and II scan DSC curves, together with the relative thermal data, are shown in the Supporting Information (Figure S1 and Table S1). PBCE shows the typical behavior of semicrystalline materials, with the endothermic glass-to-rubber transition step below room temperature at 15 °C, followed by an endothermic peak at high temperature (*T*_2_ = 168 °C), ascribable to the melting of the crystalline phase.

Copolymerization is responsible for both the reduction of *T*_2_ (from 168 °C of PBCE to 94 °C of P(BCE_65_BPripol_35_)) and ΔH_2_ (from 40 J/g of the homopolymer to 10 J/g of P(BCE_65_BPripol_35_)) as the BPripol unit amount increases. The melting endothermic phenomenon becomes also wider. All these results suggest a reduction of the crystalline portion (lower ΔH_2_) as well as the presence of crystals with a lower degree of perfection (enlarged peak and lower *T*_2_). A possible explanation of this behavior can be found in a reduction of the crystallization capability of the PBCE chains due to the insertion of Pripol subunits. In addition, a small endothermic signal (*T*_1_) is detected between *T*_g_ and *T*_2_, whose intensity remains almost constant in all of the samples. This phenomenon could be attributed to the presence of a 2-D ordered structure, arising from the stacking of aliphatic cyclohexane rings in the chair conformation, similarly to what observed for other previously studied PBCE-based systems.^31,57,58^ Its development is related to the copresence of mesogenic groups, such as the cyclohexane ring, together with flexible aliphatic units, consisting of the butylene subunits and the PE-like segments of Pripol 1009. As to the glass-transition temperatures, directly related to the amorphous phase mobility, a progressive decrease of *T*_g_ with the increase of Pripol’s amount was observed (from 15 °C of PBCE to −42 °C of P(BCE_65_BPripol_35_)). The observed trend is due to the plasticizing effect of the long aliphatic segments in the Pripol moiety. A parallel increase of Δ*C*_p_ (from 0.107 J/g°C of PBCE to 0.249 J/g°C of P(BCE_65_BPripol_35_)) occurred, indicating a progressively higher amount of amorphous fraction. It must be noticed that all the materials can be considered as rubbery at room temperature, since *T*_g_ < *T*_room_. The calorimetric profiles of the II scan, carried out after rapid cooling from the molten state, are similar to those already described for the I scan, as well as the main thermal transitions. Since in all cases an evident melting peak is present, it can be assessed that the cooling rate adopted was not high enough to block the macromolecular chains in a complete amorphous state.

Then, all of the polymers were processed in free-standing compression-molded films. After this processing, the films were stored at room temperature for 2 weeks and then characterized from the thermal, structural, wettability, and mechanical point of view.

In Figure 2B,C, I and II scan DSC curves are shown, and the relative thermal characterization data are collected in Table 2. By observation of the I scan calorimetric traces, it is clear how all the polymers are semicrystalline, with profiles similar to those of the corresponding powders. Also in this case the effect of copolymerization is visible in the decrease of both T_2_ (from 166 °C of PBCE to 98 °C of P(BCE_65_BPripol_35_)) and ΔH_2_ (from 30 J/g of the homopolymer to 4 J/g of P(BCE_65_BPripol_35_)), as well as in the widening of the melting endotherms. As to the endothermic signals (*T*_1_) between *T*_g_ and *T*_2_, they were found at about constant temperature (in a range of 41–45 °C), and their intensities slightly rise as the content of the Pripol moiety increases. Moreover, with regard to PBCE and P(BCE_95_BPripol_5_) DSC traces, multiple melting peaks are observed, ascribable either to melting/crystallization/melting phenomena or to the presence of several crystalline phases. The results obtained by wide-angle X-ray scattering analysis, shown in the following, will help to clarify this point.

The glass-transition temperatures followed the same trend already observed for the powders, with a progressive decrease with the increase of Pripol’s amount and a parallel increase of Δ*C*_p_.

A second heating scan, carried out after rapid cooling from the molten state, was performed in order to limit the crystallization of the polymers as much as possible and to record the main thermal transitions independently from the thermal history of the samples. The II scan traces (Figure 2C) turned out to be similar to those of I scan. Also in this case, the cooling rate adopted was not high enough to quench the films.

#### Structural Characterization

The compression-molded films have been studied by wide-angle X-ray scattering (WAXS), to understand the nature and the amount of crystalline phase present in the polymers under investigation. WAXS patterns are shown in Figure 2D, while the corresponding crystallinity degrees (χ_c_) are reported in Table 2. In agreement with calorimetric data, the copolymers show a decrease in the crystallinity fraction by increasing the amount of Pripol 1009. The PBCE homopolymer showed indeed the highest χ_c_ value (22%) and P(BCE_65_BPripol_35_) the lowest one (9%). All the WAXS patterns present two amorphous halos, the first more evident between 2θ = 15 and 20°, and the second much less evident between 2θ = 37.5 and 50°. As the Pripol content rises, a progressive increase of the area under the bell-shaped background line, directly related to the amorphous phase fraction, can be observed, accompanied by a reduction of the reflection intensities and sharpness, due to the decrease of the amount and quality of the crystalline phase. Previous studies^31,59^ indicated that PBCE shows polymorphism (α or β-PBCE forms), selective formation of one form being favored by copolymerization or specific thermal treatments. In this case, we can state the PBCE sample is formed essentially by β-PBCE crystals with minor amount of α-form; indeed, the most intense reflection at 19.5°, with the addition of the smaller peaks at 16.6 and 24.4°, is peculiar of β-form, whereas peaks at 15.0, 18.2, 20.7, and 22.6° are clear indication of a small amount of α-form. In the copolymers, although the low χ_c_ values make difficult to identify clearly the type of crystalline phase present, the relative amount of α-PBCE rises as the amount of Pripol increases, as evidenced by the two reflections at 18.2 and 22.4°, which become the relative most intense ones if the amorphous contribute is excluded.

#### Surface Wettability

The surface wettability of PBCE and its copolymers was evaluated by static water contact angle (WCA) measurements. The relative data are collected in Table 2, while the pictures of drops on the polymeric surfaces are collected in Figure S2. All of the materials under investigation turned out to be hydrophobic, with WCA values higher than 90°. More in detail, PBCE is the most hydrophobic among the family (101 ± 5°), while the copolymers present slightly lower WCA values (ranging from 91 ± 2 to 95 ± 3°). As a general trend, the presence, in the copolymers, of Pripol moieties characterized by a high hydrophobic character, due to the reduction of −COOR– groups per unit length, was expected to increase the polymer hydrophobicity.^46^ However, previous studies indicated that a low amount of crystalline phase determines a decrease in the hydrophobic character of the materials.^60^ Thus, to explain the observed results, we hypothesize a synergic effect of these two factors, as already seen in other copolymeric systems containing Pripol as a comonomeric unit.^47^ In this case, it is conceivable that the effect of the decrease of crystallinity results in a decrease of WCA value for P(BCE_95_BPripol_5_) and P(BCE_85_BPripol_15_). Then, by increasing the Pripol’s amount, its hydrophobic character is prevailing, or at least compensating, and although the crystallinity of the copolymers continues to decrease, the WCA values slightly increase again for both P(BCE_75_BPripol_25_) and P(BCE_65_BPripol_35_) (Table 2).

#### Mechanical Properties

**Figure 3:**
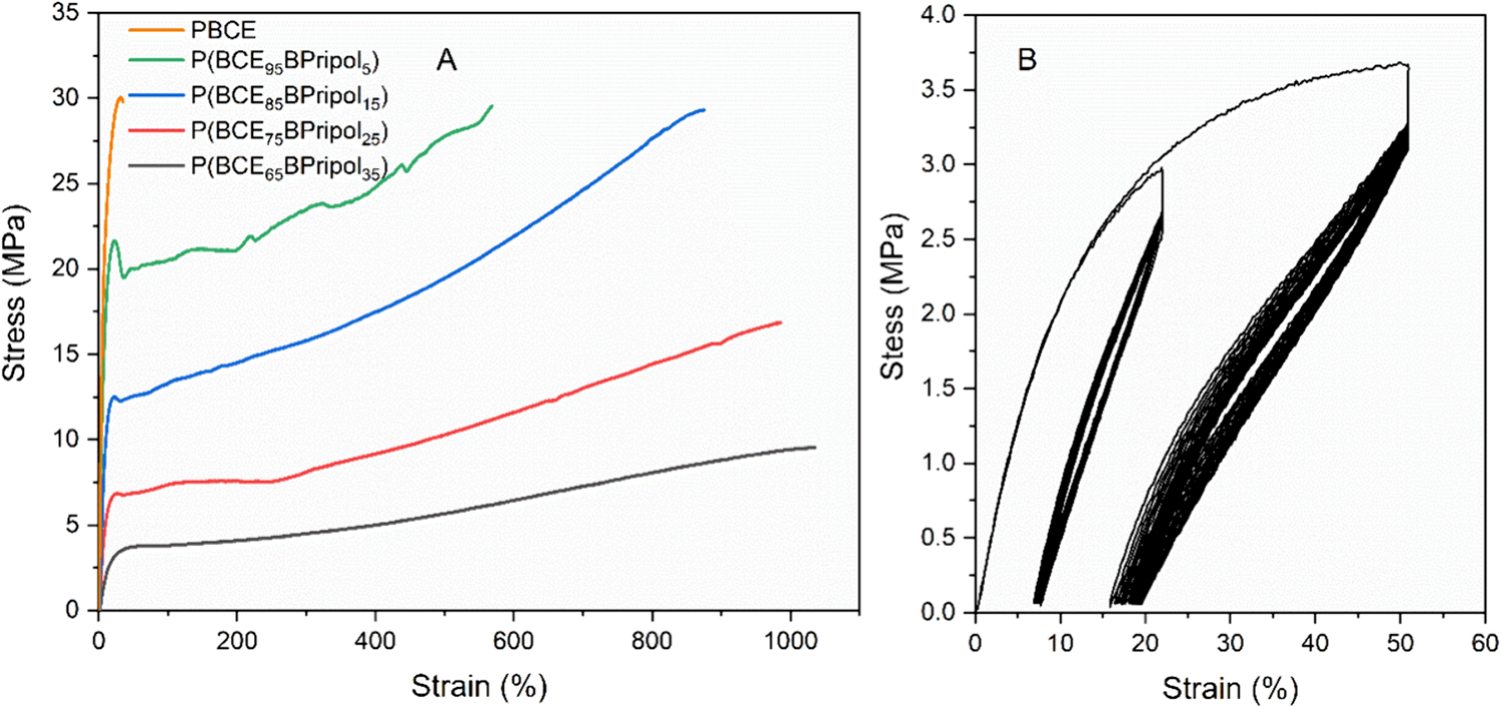
(A) Stress–strain curves of PBCE and P(BCE_*x*_BPripol_*y*_) copolymers and (B) cyclic mechanical testing of P(BCE_65_BPripol_35_) at 20 and 50% of elongation.

In order to obtain information about the mechanical properties of the synthesized materials, tensile tests were conducted on PBCE and its copolymers in the form of films. The obtained stress–strain curves are shown in Figure 3, while the mechanical characterization data (elastic modulus, *E*, stress at break, σ_B_, and strain at break, ε_B_) are reported in Table 3. The PBCE homopolymer resulted in the stiffest material, presenting the highest value of elastic modulus (560 MPa) and lowest value of elongation at break (33%). The effect of copolymerization is evident and more remarkable as the amount of comonomeric units is increased, the elastic moduli progressively decreasing and the elongations at break increasing, reaching values, for P(BCE_65_BPripol_35_), of 30 MPa and 1080%, respectively. A similar trend was also observed for stress at break, which remains high and almost constant until P(BCE_85_BPripol_15_), and then decreases. These results can be explained as due to the lowering of both *T*_g_ (i.e., higher chain flexibility) and degree of crystallinity in the copolymers with respect to the homopolymer (Table 2). Therefore, the introduction of Pripol 1009 in the PBCE main chain was effective in making the copolymers more flexible, which is a fundamental requirement for vascular applications. Particularly interesting, the low values of elastic modulus and stress at break reached by increasing the amount of Pripol are comparable to those obtained for other aliphatic polymeric systems already investigated in the literature based on PCL, PLA, and PLGA, for the treatment of occlusion of small-diameter vessels,^16,17,61−63^ and are suitable to ensure the mechanical compatibility with the surrounding vascular tissue. Moreover, the Pripol moiety, if present in sufficient quantities, can give the materials an elastomeric behavior. This last is not typical of random copolymers, as those considered in the present study, but rather is characteristic of block copolymers,^64^ in which rubbery and soft segments, responsible for the high elongation capability, are present together with hard moieties able to crystallize and thus responsible for the good elastic return. However, due to its bulky nature and high molecular mass, the Pripol 1009 subunit can be considered itself as a soft block, accountable for the elastomeric behavior and the impressive elongation at break.

**Table 3:** Mechanical Characterization Data of PBCE and P(BCE_*x*_BPripol_*y*_) Copolymers

Samples	*E*	σ_B_	ε_B_
	MPa	MPa	%
PBCE	560 ± 19	27 ± 2	33 ± 5
P(BCE_95_BPripol_5_)	356 ± 32	29 ± 4	550 ± 147
P(BCE_85_BPripol_15_)	175 ± 19	28 ± 3	979 ± 160
P(BCE_75_BPripol_25_)	89 ± 12	17 ± 1	1003 ± 156
P(BCE_65_BPripol_35_)	30 ± 2	8 ± 2	1080 ± 282

As a result, although the copolymers containing 25 mol % of Pripol or less are characterized by the presence of yielding, which is detectable already at low elongations, P(BCE_65_BPripol_35_) did not present this phenomenon (Figure 3). For this reason, on this copolymer, cyclic stress–strain measurements were performed by applying two different elongations, 20 and 50%, for 20 cycles. In both cases, the films showed quite good recovery of about 66%. The recovery is not complete since once the original microstructure is disrupted during the first cycle, and the sample does not have enough time to completely recover before the following cycle starts. In parallel, a decrease of hysteresis energy (*U*_hys_), by increasing the number of cycles, was observed. For the elongation at 20%, *U*_hys_ ranged from 19 (±4) hJ/mm^3^ in the first cycle to 2.2 (±0.1) hJ/mm^3^ for the other cycles, while for the elongation at 50%, it varied between 92 (±19) and 9 (±1) hJ/mm^3^, indicating an increase of energy recovery with the number of cycles.

### Stability Performance of the Materials

#### Hydrolytic Degradation Tests

In order to evaluate the material stability in an environment mimicking the human body (pH 7.4) under both physiological (37 °C) and accelerated (70 °C) conditions, which is a very important property for long-term applications in the biomedical field, hydrolytic degradation tests were carried out.

**Figure 4:**
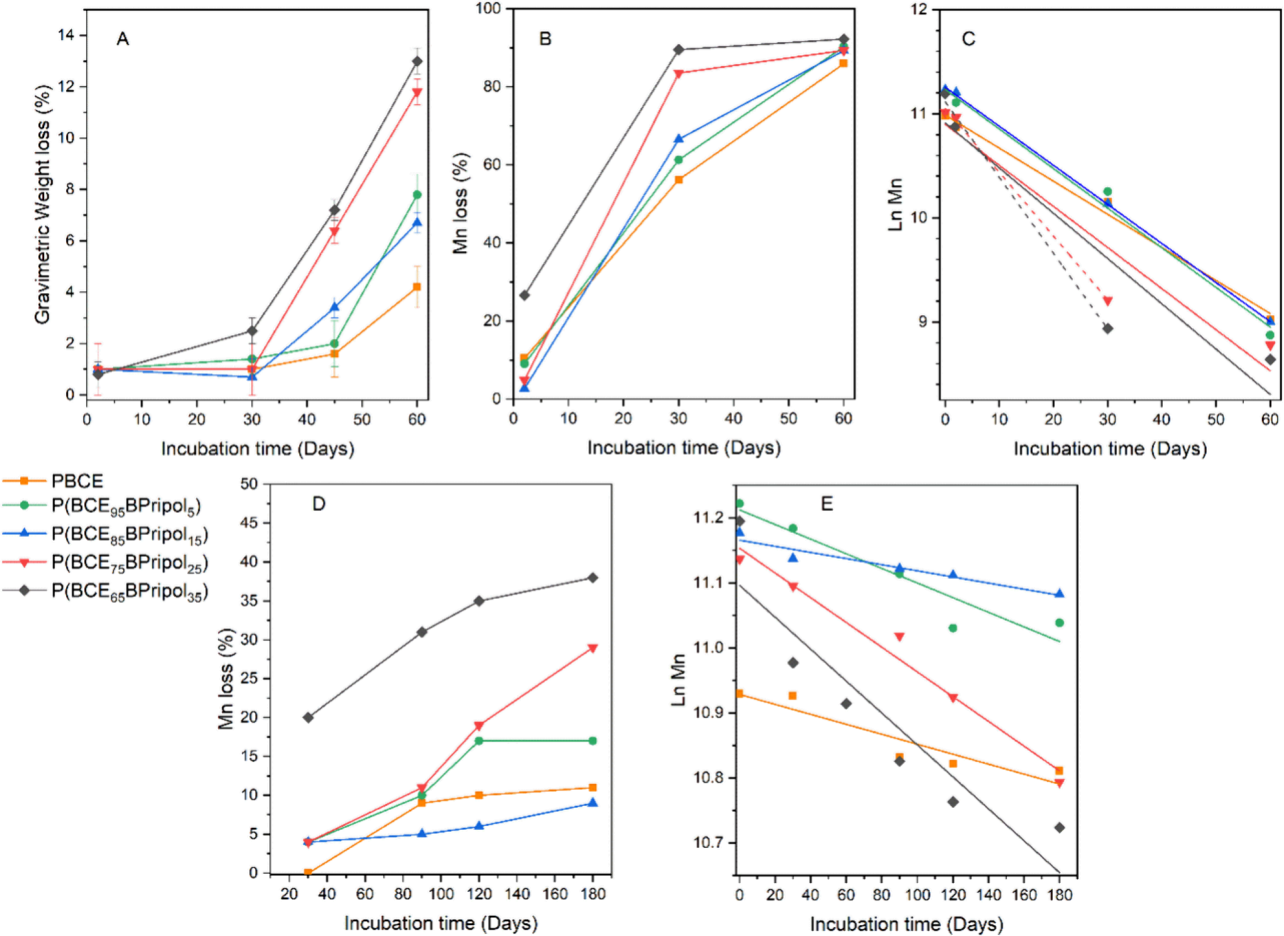
(A) Gravimetric weight loss, (B) molecular weight loss, and (C) natural logarithm of molecular weight of PBCE and P(BCE_*x*_BPripol_*y*_) copolymers after hydrolytic degradation tests under accelerated condition (70 °C). (D) Molecular weight loss and (E) natural logarithm of molecular weight of PBCE and P(BCE_*x*_BPripol_*y*_) copolymers after hydrolytic degradation tests under physiological conditions (37 °C).

Figure 4A shows the percentage gravimetric weight loss until 60 days of incubation at 70 °C. After 2 and 30 days, the weight loss of all of the polymers was negligible and comparable. After 45 days of incubation, some differences started emerging: in detail, PBCE and P(BCE_95_BPripol_5_) have both jetted a negligible weight loss, lower than 2%, and weight loss of P(BCE_85_BPripol_15_) was of about 3%, while P(BCE_75_BPripol_25_) and P(BCE_65_BPripol_35_) at this time point lost about 7%. This trend was even more pronounced after 60 days: PBCE showed a stability to degradation higher than that of its copolymers (final weight loss of about 4%), while the two copolymers richest in Pripol 1009 showed the highest weight loss, of about 14%. This result is not surprising considering that PBCE is the most crystalline and hydrophobic material, with the highest *T*_g_. As known, more flexible chains (lower *T*_g_) and/or the presence of amorphous regions, more accessible to water molecules, are those first attacked during hydrolysis.^65,66^ In addition, hydrophilic materials are easier to be attacked due to their higher affinity to water.^67^ However, it must be noticed that in all cases, the overall weight loss at the end of experiment was limited, indicating a good long-term stability.

As it is known, hydrolysis is a bulk phenomenon, meaning that at the beginning, the macromolecular chains attacked by water start breaking, resulting in a decrease in the molecular weight. In parallel, the gravimetric weight remains almost unaltered, as the hydrolyzed chains are not small enough to solubilize. For this reason, to follow the degradation process during its initial stages, the percentage of molecular weight loss, calculated by means of GPC, was also measured. From the data reported in Figure 4B, it can be observed that after 2 days all the materials, except P(BCE_65_BPripol_35_), present a low molecular weight loss, in line with those of other aliphatic polymeric systems investigated in the literature for the same purposes.^17^ After 30 days, this loss rapidly increases as the Pripol’s amount present in the copolymers increases: from PBCE to P(BCE_85_BPripol_25_), the loss is within 56 and 67%, while for P(BCE_75_BPripol_25_), it is more than 80%. After 60 days of incubation, degradation occurs at greater extent, the molecular weight loss reaching values between 86 and 89%. Conversely, P(BCE_65_BPripol_35_) starts losing molecular weight already after 2 days (weight loss of about 27%), reaching 90% loss after only 30 days. The fastest loss of molecular weight in the case of P(BCE_65_BPripol_35_) can also be ascribed to its low melting temperature (Table 2), close to the testing temperature (70 °C).

Thus, by comparing the gravimetric and the molecular weight losses, it is possible to notice that in this latter case, the decrement was more remarkable and, as a general trend, the gravimetric weight loss begins after 30 days, when the residual molecular weight is less than 50%, confirming the bulky nature of the degradation process.

In physiological conditions (37 °C), gravimetric weight loss remains in all cases below 1%, while the molecular weight decrease is lower than the one observed under accelerated conditions, as shown in Figure 4D. In this case, *M*_n_ loss is lower than 20% from PBCE to P(BCE_85_BPripol_15_), ranging between 30 and 37% for the copolymers richest in Pripol 1009, with a trend in line with that previously observed for the studies carried out at 70 °C.

In Figure 4C,E, the natural logarithm of molecular weight is shown as a function of incubation time. From these curves, it was possible to derive the kinetic constant (*k*) of degradation (Table 4), which is a parameter that determines how fast materials will degrade in a certain environment.^68,69^

**Table 4:** Hydrolysis Kinetic Constants (*k*) of PBCE and P(BCE_*x*_BPripol_*y*_) Copolymers under Accelerated and Physiological Conditions

	**Accelerated Conditions** (70 °C)				**Physiological Conditions** (37 °C)	
			**until 30 days**			
	***k***	***R***^**2**^	***k***	***R***^**2**^	***k***	***R***^**2**^
**Samples**	**days**^**–1**^ **× 10**^**–3**^		**days**^**–1**^ ****×** 10**^**–3**^		**days**^**–1**^ **x 10**^**–3**^	
PBCE	31 ± 2	0.9914			0.8 ± 0.2	0.8214
P(BCE_95_BPripol_5_)	38 ± 3	0.9898			1.1 ± 0.2	0.8555
P(BCE_85_BPripol_15_)	37.5 ± 0.5	0.9960			0.5 ± 0.1	0.9065
P(BCE_75_BPripol_25_)	40 ± 9	0.9138	61 ± 2	0.9988	1.9 ± 0.2	0.9065
P(BCE_65_BPripol_35_)	43 ± 12	0.8750	73 ± 5	0.9955	2.5 ± 0.5	0.8167

Under accelerated conditions, the influence of the materials’ crystallinity on the kinetics of hydrolysis seems to play a key role. In fact, PBCE showed the smallest kinetic constant, which is very similar to those of P(BCE_95_BPripol_5_) and P(BCE_85_BPripol_15_). For the other two copolymers, the trend is different: indeed, for P(BCE_75_BPripol_25_) and P(BCE_65_BPripol_35_) two steps can be seen, the former is related to the stage in which the materials witness a drop in molecular weight without any gravimetric weight loss, and the latter is starting when also gravimetric weight loss occurs. During the first phase of degradation, according to studies previously carried out in the literature, the hydrolysis process is accelerated due to the autocatalytic effect exerted by short polymeric chains in the sample.^70^ On the contrary, the second step starts when the gravimetric weight loss begins (after 30 days), as a result of the diffusion of oligomers from the polymeric matrix to the incubation medium, as already noted by Rogriguez et al. in the case of PLA.^70,71^

In physiological conditions, all of the kinetic constants are very small, with the two copolymers richest in Pripol being characterized by the highest *k*. Surprisingly, the value of P(BCE_85_BPripol_25_) is the smallest and very close to that of PBCE, suggesting a not completely linear behavior, which will be the object of further investigations.

**Figure 5:**
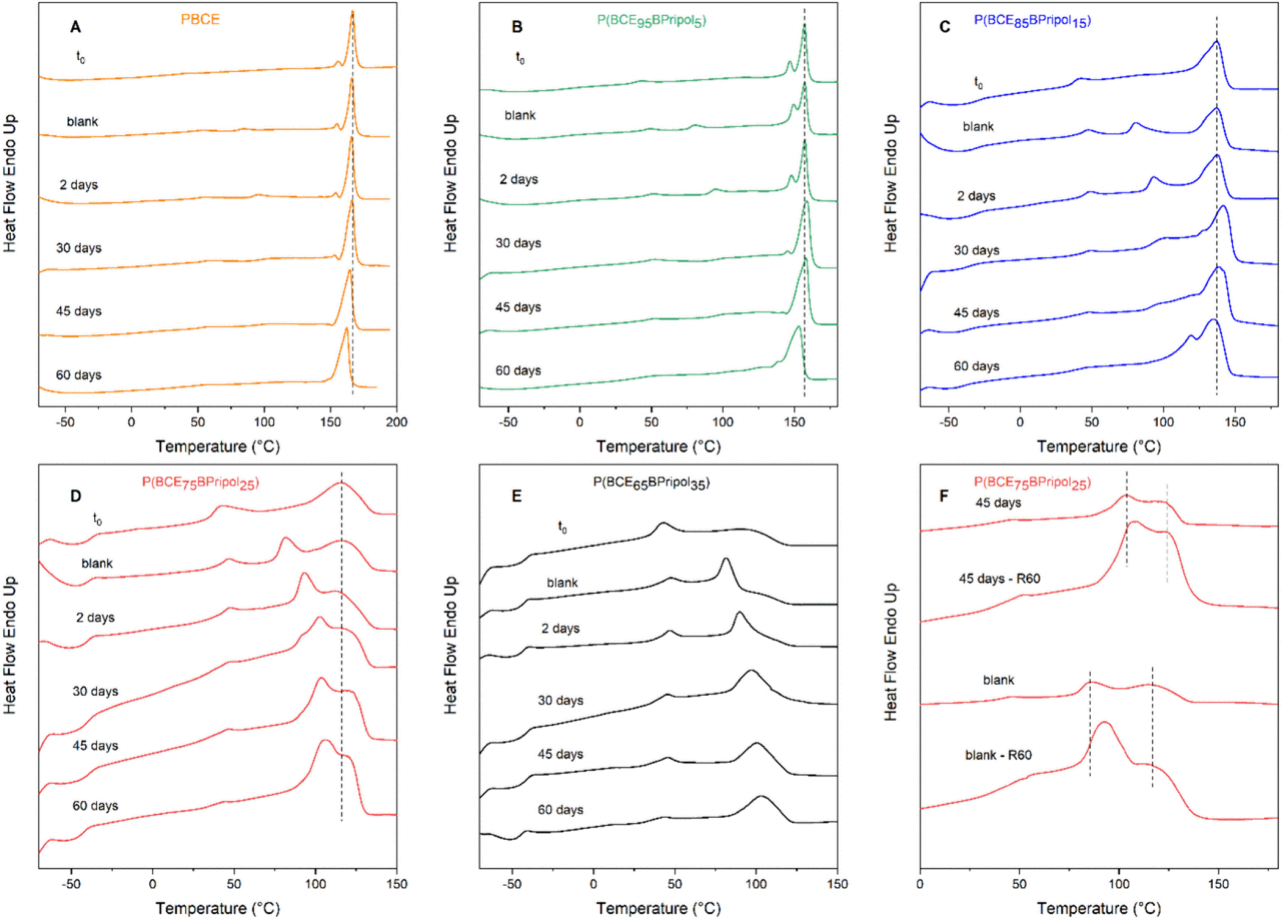
(A–E) I DSC scans of PBCE and P(BCE_*x*_BPripol_*y*_) films before and after hydrolytic tests at 70 °C, together with those of blanks. (F) Comparison of DSC traces at different scan rates of 20 °C/min (R20) and 60 °C/min (R60) for P(BCE_75_BPripol_25_) partially degraded film and blank.

The partially degraded materials were also subjected to the DSC analysis to check whether any difference in the main thermal transitions occurred. In Figure 5, the I scan DSC traces at different time points are shown, together with those of their relative blanks (samples incubated at 70 °C but without phosphate buffer), while the relative thermal data are listed in Table S2. For PBCE and P(BCE_95_BPripol_5_), DSC profiles of blanks remain constant and similar to the one of the original film during the whole test, indicating that the incubation temperature does not determine an evolution of crystallinity. As to partially degraded samples, a different trend can be observed, as for PBCE the hydrolysis first involves the amorphous phase (higher Δ*H*_m_ from day 30), and then, after 60 days, it starts involving also the crystals. Indeed, at this time point, the melting peak becomes wider and slightly shifted toward lower temperatures (Table S2). In the case of P(BCE_95_BPripol_5_), the shape of the melting peak changed starting from the 30th day of incubation from a double sharp peak to a single enlarged one. Moreover, after 60 days of incubation, the main melting phenomenon resulted in a shift toward lower temperature (Table S2). These evolutions can be explained as due to the development of oligomers with different length and high crystallization capability that melt at lower temperatures (enlarged melting peak and lower *T*_m_) and to the attack, by water molecules, of the amorphous phase (higher Δ*H*_m_).

For P(BCE_85_BPripol_15_), the testing temperature does not determine the evolution of the main melting peak of the blank sample over time. The calorimetric profile, compared to the one of the neat sample, only shows a slight additional annealing peak, which becomes evident around 80 °C, a temperature close to the incubation one. This endothermic phenomenon can be observed also in the partially degraded samples, becoming less pronounced and shifted toward higher temperatures over time, indicating that the hydrolytic attack regarded, at least during the first phases of degradation, the less perfect low-melting crystalline phase. The formation of higher amount of crystalline phase is even clearer after 45 days, and then after 60 days of incubation, *T*_m_ decreases, and the melting endotherm shows a double peak. Compared to the blank sample, the melting enthalpy was almost the same until 30 days of incubation and more than doubled after 45 and 60 days (Table S2). Probably, in this last phase, the hydrolytic attack involved not only the amorphous phase (higher Δ*H*_m_) but also the high-melting crystalline phase (double melting peak and lower *T*_m_).

In the case of P(BCE_75_BPripol_25_), analogously to the previous samples, no evolution of the calorimetric profile of the blank samples over time was not observed. By comparing this profile to the one of the neat sample, the presence of a further intense melting peak at 85 °C was observed, due to annealing at incubation temperature. This peak becomes more intense over time and progressively shifted toward higher temperatures in the hydrolyzed samples and, starting from 30 days of incubation, is partially overlapped to the main melting phenomenon. This additional peak could be attributable to the presence of a 2-D ordered phase, as already observed in nonhydrolyzed samples. To shed light on the nature of this low-temperature endothermic peak, a DSC scan was carried out at a rate of 60 °C/min (R60) on a sample recovered after 45 days of hydrolysis and on the blank. As observable in Figure 5F, in both cases, the different heating rate did not affect the position of the higher melting peak, as expected, but was responsible for a shift to higher temperatures of the lower melting peak, which becomes even more evident than the higher melting one. As is well-known, melting is a first-order transition, and consequently, it does not depend on the heating rate, while second-order transitions, such as the isotropization of 2-D ordered phases, are strictly dependent on the scanning rate. Therefore, the obtained result can be due to (1) the attack of amorphous phase by water molecules, which permits the less perfect crystalline phase to reorganize in a more perfect one, and/or (2) the presence of a 2-D ordered phase, which improves during the permanence at 70 °C.

For P(BCE_65_BPripol_35_), the DSC profile of the blank sample, which remains constant over time, compared to the one of the neat sample, shows a lower melting phenomenon around 47 °C, followed by an additional annealing peak around 80 °C. As to partially degraded samples, a similar trend can be observed, the most intense endothermic peak shifting from 98 to 103 °C and becoming more intense over time (Table S2). To explain this behavior, it is important to consider that the temperature at which hydrolytic tests were performed is very close to the beginning of melting phenomenon: for this reason, the formation of more perfect crystals over time is possible, thanks to erosion of the amorphous phase.

The DSC analysis was performed also on films after hydrolytic degradation tests at 37 °C. In Figure S3, I scan DSC traces at different time points are shown, together with those of the blanks (samples incubated at 37 °C but without phosphate buffer), while the relative thermal data are listed in Table S3. For all the materials investigated, DSC profiles of blanks remain constant and similar to the ones of the original films during the whole test, indicating that the incubation temperature does not determine an evolution of crystallinity. The only exception is represented by the shift of the low-melting endotherm toward higher temperature (from 41 to 45 to 55–61 °C) due to the incubation at 37 °C.

Additionally, any remarkable changes in DSC profiles cannot be detected in partially degraded samples, compared to those of the relative blanks after 30 as well as 180 days of incubation, confirming how the permanence in the physiological environment did not alter the main thermal transitions of the studied materials.

### Texture Features and Surface Zeta Potential Properties

**Figure 6:**
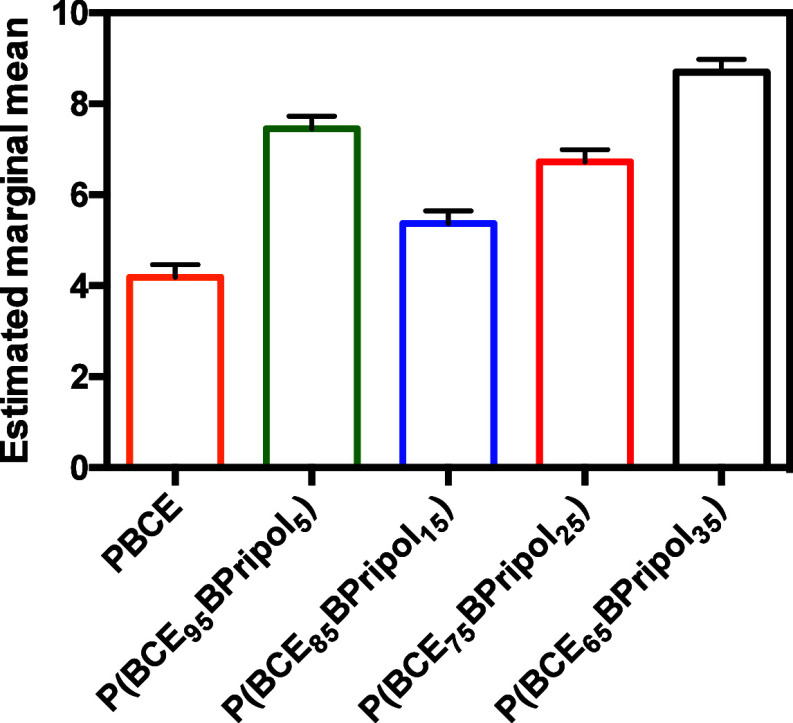
Estimated marginal means of the biomaterials. All comparisons are significant (*p* < 0.05) except P(BCE_75_BPripol_25_) vs P(BCE_95_BPripol_5_).

The surface texture of the biomaterials was evaluated by estimating the marginal means of the 21 Haralick’s texture features (Figure 6). All comparisons are significant (*p* < 0.05) except P(BCE_75_BPripol_25_) vs P(BCE_95_BPripol_5_).

Of note, an estimated marginal mean, in a model with covariates (in this work, the Haralick’s texture features), is a predicted mean, one for each case (in this work, the biomaterial type), calculated at the mean of the covariates in order to give fairer comparisons.

**Figure 7:**
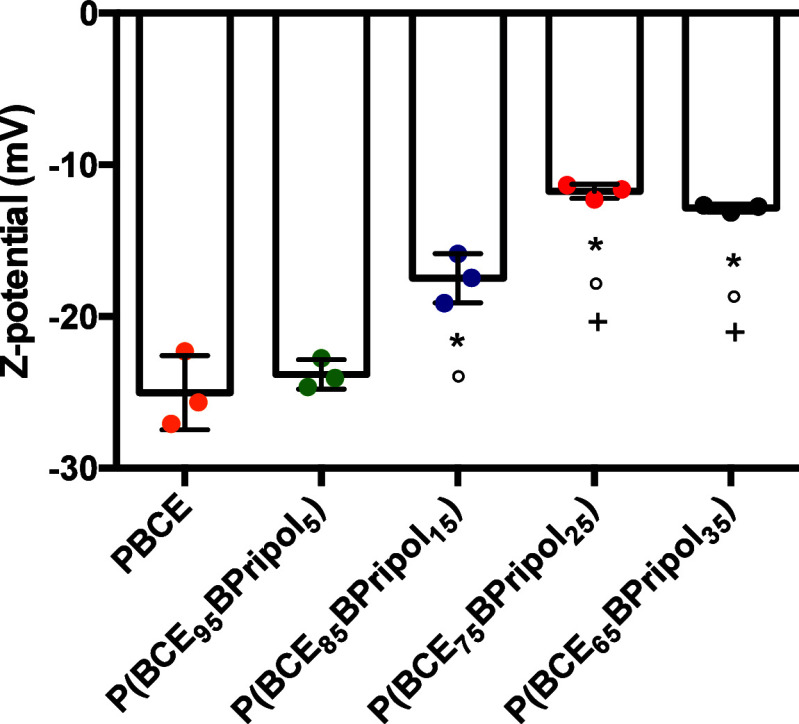
*Z*-Potential assessment at pH 7.4. Results are presented as mean ± SD (*N* = 3; symbols indicate statistical significance vs PBCE (*), vs P(BCE_95_BPripol_5_) (°) and vs P(BCE_85_BPripol15_5_) (^+^)).

All the films were also characterized by *Z*-potential measurement. The graph in Figure 7 represents the average zeta potential of PBCE and its copolymers. A decrease in *Z* potential value was observed by increasing the amount of Pripol. As expected, the high hydrophobic and nonpolar counit, consisting of long PE-like moieties both in the main and in the side chains, is responsible for values of zeta potential closer to zero. Conversely, the more negative *Z*-potentials observed for both PBCE homopolymer and P(BCE_95_BPripol_5_) can be attributed to the effect of delocalization of the negative charges of the ester bonds, whose amount per chain unit is higher in these two materials, in agreement with literature data.^72−74^

### In Vitro Biological Properties

**Figure 8:**
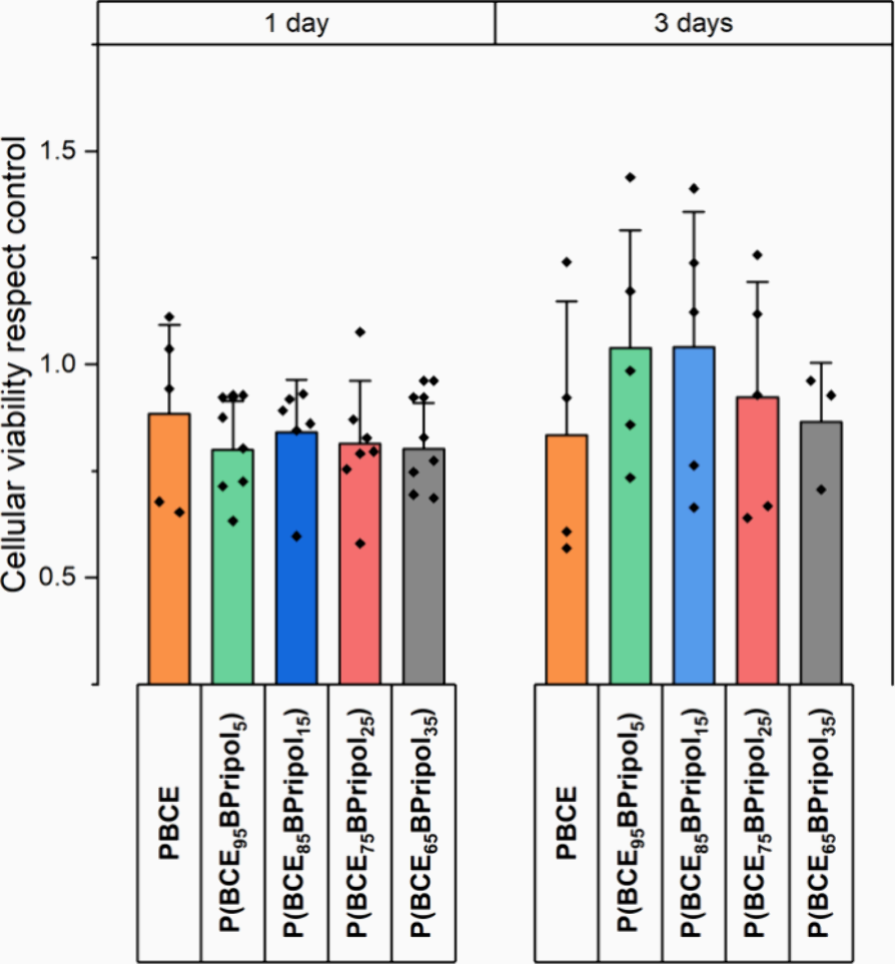
Cell viability of PBCE and P(BCE_*x*_BPripol_*y*_) copolymers with respect to the control after 1 and 3 days. The data are presented as mean ± SD.

In order to evaluate the applicability of all these materials in vascular applications as small blood vessel substitutes, direct-contact in vitro cytotoxicity tests were carried out, using human umbilical vein endothelial cells (HUVECs) due to its wide use in cardiovascular and clinical research as an endothelial model.^75^ In Figure 8, the cell viability for all the materials after 1 and 3 days of incubation is reported compared to the control (cells cultured in a medium without the presence of the polymeric film). Considering that a material is cytotoxic if its cellular vitality is less than 70% compared to the control,^76^ after 1 and 3 days, all the materials under study turned out to be not cytotoxic, with vitality values all comparable and ranging between 80 and 90%. Therefore, from these preliminary results, it is possible to assess that all the materials turned out to be not cytotoxic and suitable for applications in contact with cells.

In terms of hemocompatibility, the surfaces of the biomaterial were exposed to blood components to provide evidence of the biomaterial’s potential as a blood-contacting device. Contact with blood component can trigger several complex biochemical reactions such the activation of the coagulation process.^77^ The effects of the biomaterials on the coagulation process were then tested by PT and aPTT measurements. PT and aPTT were selected as reliable measures of the ability of blood to coagulate through extrinsic and intrinsic coagulation mechanisms, respectively.^78^ As shown in Figure S4A, all materials tested had no impact on PT (Figure S4A) and aPTT (Figure S4B) clotting times, indicating that the extrinsic and intrinsic pathways were unperturbed by the surfaces generated. The values obtained were indeed similar to those observed for human plasma incubated in the test tubes without any materials (CTRL), which values within normal clinical reference ranges (PT and APTT reference values 10–12 and 20–32 s, respectively) as provided by the laboratory where the analyses were carried out. These values are highly comparable to scaffolds previously designed for vascular applications: Wang et al. reported similar values on commercial chitosan-based materials (PT of 8.0 s and APTT of 30 s) and on nanofibers with a PLA/chitosan core and shell (PT of 9.2 s and APTT of 32 s).^79^

**Figure 9:**
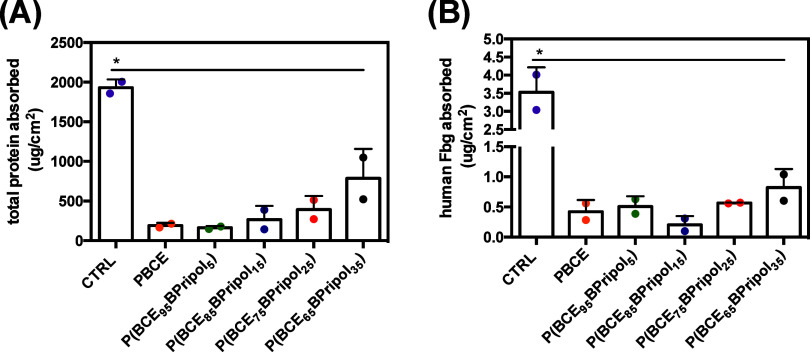
Total plasma proteins (A) and human fibrinogen (B) absorption in the different surfaces from human plasma after 1 h of incubation at 37 °C and determined as described in the Materials and Methods section. Data are presented as mean ± SD (*N* = 2). CTRL represents the absorption of (A) total protein or (B) hFbg on high binding well plates. Symbol (*) indicates statistical significance vs CTRL (*: *p* < 0.05).

When an artificial material encounters a biological fluid, such as blood, the soluble proteins it contains quickly adsorb and form a monolayer on the surface. This nonspecific protein–surface interaction is called fouling and is relevant problem in medical applications.^77,80^ Adsorbed proteins can lead to the activation of a series of events such as complement activation^81^ and the coagulation cascade,^82^ which can be responsible for thromboembolic complications with serious or even life-threatening outcomes. Fbg is one of the major adsorbed proteins in human blood and adsorbs very rapidly to the surface of almost all biomaterials in contact with biological fluids such as blood plasma.^83^ Absorbed Fbg promotes platelet and monocyte-macrophage adhesion, which plays a key role in blood coagulation and foreign body reactions associated with biomaterial implants. To test the ability to bind plasma proteins, the surfaces were incubated with human plasma and the adsorption of total protein and human Fbg was determined using the BCA protein assay (Figure 9A) and the ELISA assay (Figure 9B), respectively. In terms of total plasma protein, a significantly lower adsorption of total proteins on the surfaces was observed when comparing the results with the positive control (CTRL) (Figure 9A). About the absorption of hFbg, the results in Figure 9B show that for the surfaces studied in this work, the amount of hFbg absorbed by human plasma on the material surfaces was significantly lower than that determined in the control.

It is worth noting that the amount of hFbg adsorbed on all surfaces was less than 0.70 μg/cm^2^, which is much lower than that observed for other blood-contacting devices, such as the poly(ester-urethane) film containing phosphorylcholine (PC) groups on the side chains (hFbg adsorption of around 2–0.60 μg/cm^2^, depending on PC content), suggesting a poor protein adsorption capacity.^84^ Furthermore, similar to what was found for total protein adsorption, no significant difference between the materials tested was detected (Figure 9A). This trend in Pripol-dependent fibrinogen absorption was also observed when a human fibrinogen solution was incubated directly on the surfaces and quantified by ELISA (Figure S5). It can be concluded that these data are consistent with findings in the literature, showing that surface chemistry plays an important role in determining absorption. However, additional investigations would be necessary to understand whether the structures analyzed here act by influencing not only absorption but also the type of conformation (pro- or antiplatelet adhesion) that the adsorbed fibrinogen may adopt on the surface of materials.^82,85,86^

**Figure 10:**
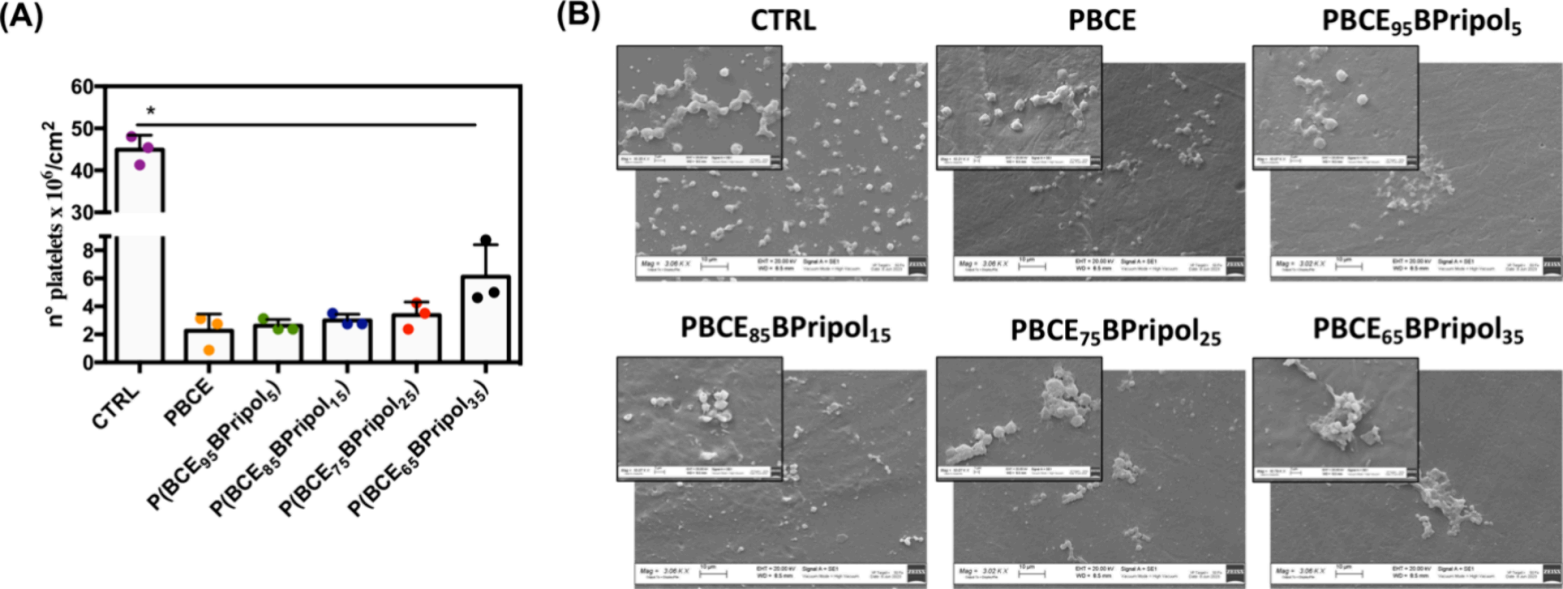
Adhesion of platelets (PLT) obtained from Human platelet-rich plasma (hPRP) after 1 h at 37 °C with different films. (A) Determination of human PLT adhesion by LDH assay. Symbol (*) indicates statistical significance vs CTRL (TCPS) (*: *p* < 0.05). (B) Representative SEM images of PLT on the different samples. Images were acquired at 3k× magnification (scale bar 20 μm), and insets at 10k× (scale bar 2 μm). No pseudopodia, characteristics of activated PLTs, was observed on either the control or the samples. CTRL: thermanox.

As an additional exploration of the materials’ hemocompatibility, their interaction with blood platelets was also studied. Blood platelets are specialized fragments of megakaryocytes that are considered key elements in the thrombus formation process.^87^ Therefore, the interaction of platelets with the surface of biomaterials in contact with blood is crucial for understanding the thrombogenicity of a material and subsequently for assessing its success or failure in vascular engineering.^88^ Platelet-biomaterial contact was then assessed by quantitative (LDH assay) and qualitative (SEM) determination of human platelet adhesion obtained after the incubation of PRP in each manufactured material. Figure 10A shows the number of adhered platelets on the test surfaces evaluated by the LDH assay (platelets/cm^2^). The adhesion of platelets on all materials was statistically different from TCPS, which showed the highest number of platelets/cm^2^ (*p < 0.05). The materials tested showed less platelet adhesion (Figure 10A) (approximately <10% compared to the number of platelets seeded), which confirms that material characteristics are critical in determining the interaction with platelets. SEM images confirmed these findings (Figure 10B). In the controls, a higher number of predominantly spherical platelets were observed, homogeneously distributed. In contrast, a different distribution of platelets was observed on the materials: a low number of platelets was observed on all materials, and as Pripol 1009 content increased, the few adherent platelets were mainly found on more aggregated structures. It should also be noted that, like other medical polyesters for vascular tissue engineering^89,90^ no activation of the platelets was observed on either the control or the samples, as no change in shape associated with their activation (development of small pseudopodia on the surface of the adherent platelets) was observed, which could be an indication that these materials may have potential antithrombotic properties.

## Conclusions

A new family of random cycloaliphatic copolymers based on PBCE and containing a Pripol 1009 moiety characterized by a six membered aliphatic ring as well and long aliphatic ramifications and PE-like segments have been successfully synthesized to evaluate their possible use for the treatment of CVDs and, in particular, the occlusion of vessels with small diameter. To this aim, the chemical design was optimized to modulate the physical–chemical properties of the starting homopolymer. In detail, through copolymerization, it was possible to reduce PBCE crystallinity and stiffness, the main limitations for its applications in soft tissue engineering, while maintaining at the same time, its thermal stability, which is one of its strong points. Such results were supported by DSC and WAXS techniques, which indicated that by increasing the amount of the counit, a decrease of both glass-transition temperature, melting temperature, and melting enthalpy, as well as crystallinity degree, can be obtained due to the internal plasticizing effect of the long aliphatic ramifications and PE-like segments in the Pripol moiety. Copolymerization allowed one to obtain stable materials under physiological conditions for long times, also characterized by a flexible mechanical response, with elastic modulus and strength at break comparable to those of other polymeric systems already investigated in the field of vascular tissue engineering. Moreover, in the copolymer containing the highest amount of Pripol, a thermoplastic elastomeric behavior was observed.

Thus, according to the obtained results, copolymerization was revealed to be a very effective tool for fine-tuning PBCE properties, allowing to achieve, at the same time, the proper mechanical characteristics for vascular tissue engineering and surface properties, suitable for cell growth. For blood-contacting biomaterials, hemocompatibility is also a key parameter. The very low platelets adhesion as well as the almost negligible interaction with other blood components can be ascribed to the synergistic effect of both peculiar surface chemistry^91^ and the quite hydrophobic character^92,93^ of these materials.

Although it is difficult to discuss an actual improvement in biological performance compared to other vascular engineering materials due to the experimental biological variability found in the literature (values vary slightly between laboratories depending on the equipment and methods used, in vitro vs in vivo models), preliminary in vitro cytotoxicity and hemocompatibility assays have demonstrated the feasibility of these PBCE materials for the fabrication of vascular tissue engineering devices. For the clinical relevance of our results, however, further studies are required.

## Supporting Information Available

I and II DSC scans of PBCE and P(BCE_*x*_BPripol_*y*_) films in form of purified powders, together with the relative thermal data, pictures of water drops on films, thermal characterization data of films before and after hydrolytic tests at 70 and 37 °C, I DSC scans of films before and after hydrolytic tests at 37 °C, PT and APTT values of the platelet poor plasma (PPP) in contact for 1 h at 37 °C with the different films, and absorption of a solution of human fibrinogen (hFbg) on the different films and assessed through an antifibrinogen HRP-conjugated antibody and quantified via ELISA assay (PDF)
