# Supplementary material for: Synthesis of Flexible Random Copolymers of Poly(butylene trans-1,4-ciclohexanedicarboxylate) Containing Pripol Moiety as Potential Candidates for Vascular Applications: Solid-State Characterization and Preliminary In Vitro Biocompatibility and Hemocompatibility
Source: Biomacromolecules. 2025 Apr 30;26(5):2882–99. doi: 10.1021/acs.biomac.4c01668 (PMC12076511; doi:10.1021/acs.biomac.4c01668)
Supplement: Supplementary file 1 — bm4c01668_si_001.pdf [file bm4c01668_si_001.pdf]

Supplementary Information

Synthesis of flexible random copolymers of poly(butylene trans-1,4-ciclohexanedicarboxylate) containing Pripol moiety as potential candidates for vascular applications: solid state characterization and preliminary *in vitro* biocompatibility and hemocompatibility

Edoardo Bondi (a), Nora Bloise (b, c, d), Michelina Soccio (a), Giulia Guidotti (a), Ilenia Motta (e) †, Massimo Gazzano (f), Marco Ruggeri (g), Lorenzo Fassina (h), Emilia Genini (i), Livia Visai (b, c, d), Gianandrea Pasquinelli (e, j), Nadia Lotti (a)*

a) Department of Civil, Chemical, Environmental, and Materials Engineering, University of Bologna, Via Terracini 28, Bologna, 40131, Italy

b) Molecular Medicine Department (DMM), Centre for Health Technologies (CHT), Unità di Ricerca (UdR) INSTM, University of Pavia, 27100 Pavia, Italy

c) UOR6 Nanotechnology Laboratory, Department of Prevention and Rehabilitation in Occupational Medicine and Specialty Medicine, Istituti Clinici Scientifici Maugeri IRCCS, Via Maugeri 4, 27100 Pavia, Italy

d) Interuniversity Center for the Promotion of the 3Rs Principles in Teaching and Research (Centro 3R), Operative Unit (OU) of University of Pavia, 27100 Pavia, Italy

e) Department of Medical and Surgical Sciences (DIMEC), University of Bologna, Via Massarenti 9, Bologna, 40138, Italy

f) Institute for Organic Synthesis and Photoreactivity, ISOF-CNR, Via Gobetti 101, Bologna, 40129, Italy

g) Department of Drug Sciences, University of Pavia, Viale Taramelli 12, 27100 Pavia, Italy

h) Department of Electrical, Computer and Biomedical Engineering, University of Pavia, Via Ferrata 5, 27100 Pavia, Italy

i) Fondazione IRCCS Policlinico San Matteo, 27100, Pavia, Italy

j) Pathology Unit, IRCCS Azienda Ospedaliero-Universitaria di Bologna, Bologna, 40138, Italy

† Present address: Alma Mater Institute on Healthy Planet, University of Bologna, Bologna, 40138, Italy

*Email: giulia.guidotti9@unibo.it; ORCID ID: 0000-0001-6879-2989

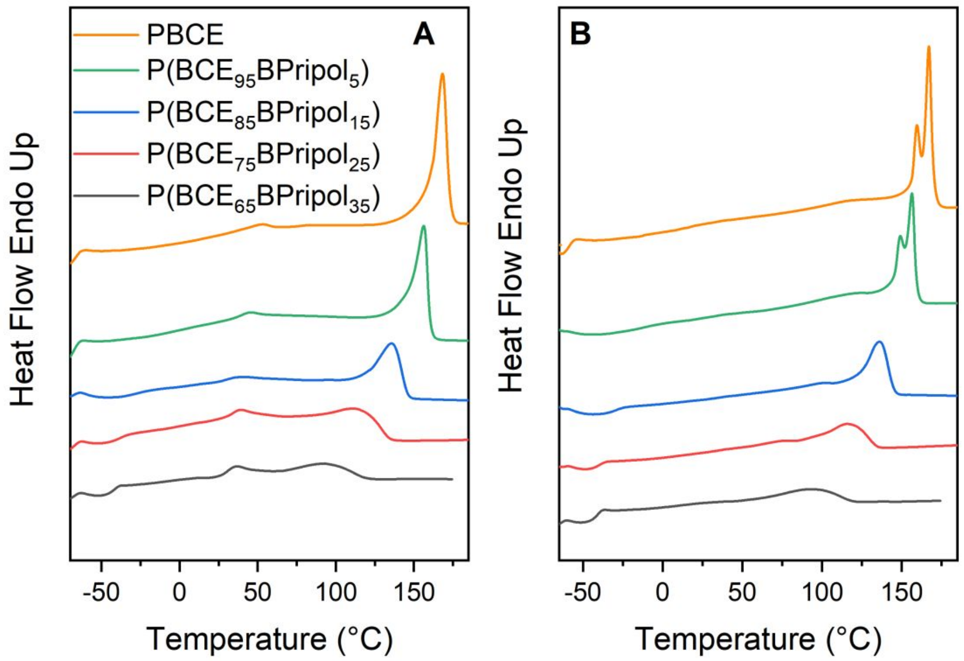

Figure S1. A) I and B) II DSC scans of PBCE and P(BCE_xBPripol$_y$) films in form of purified powders.

Table S1. Thermal characterization data (DSC) of PBCE and P(BCE_xBPripol$_y$) purified powders.

Sample	I Scan						II Scan			
	T_g	Δc_p	T_1^*	ΔH_1^*	T_2^*	ΔH_2^*	T_g	Δc_p	T_2^*	ΔH_2^*
	˚C	J/g·˚C	˚C	J/g	˚C	J/g	˚C	J/g·˚C	˚C	J/g
PBCE	15	0.107	54	2	168	40	11	0.086	167	31
P(BCE_{95}BPripol$_5$)	-11	0.102	44	1	156	31	-9	0.069	156	23
P(BCE_{85}BPripol$_{15}$)	-26	0.165	38	2	136	23	-29	0.195	136	19
P(BCE_{75}BPripol$_{25}$)	-38	0.205	39	2	115	15	-40	0.170	117	13
P(BCE_{65}BPripol$_{35}$)	-42	0.249	36	1	94	10	-42	0.274	94	7

* T_1: temperature of isotropization; T_2: temperature of melting; ΔH_1: enthalpy of isotropization; ΔH_2: enthalpy of melting

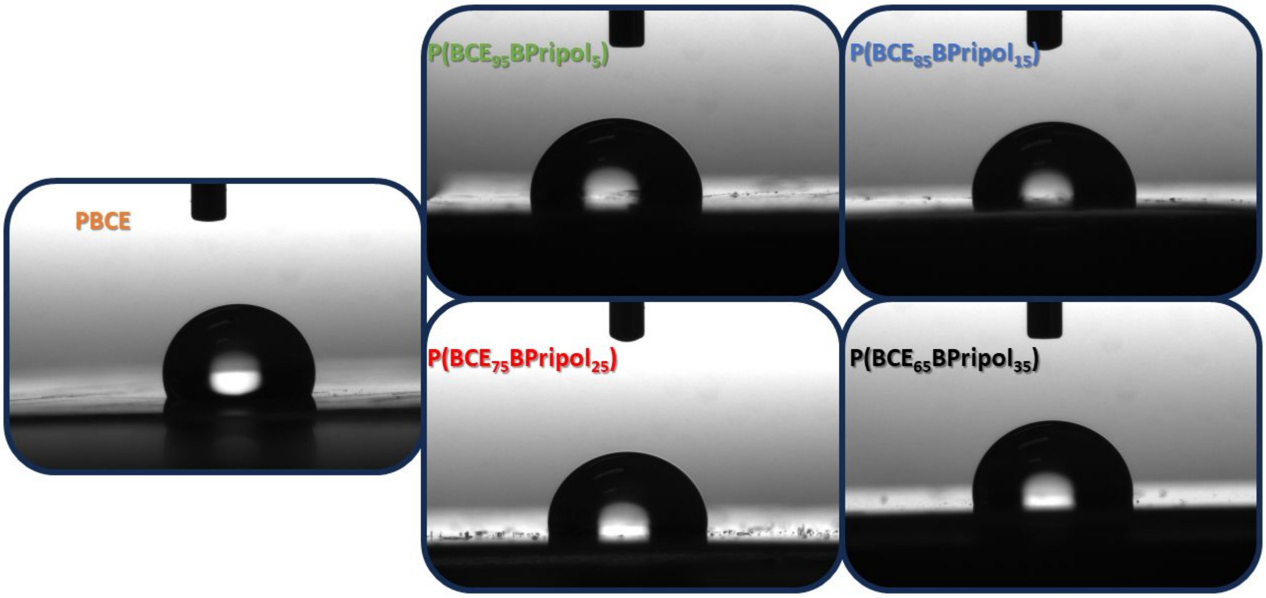

Figure S2. Pictures of water drops on PBCE and P(BCE_xBPripol$_y$) copolymeric films.

Table S2. Thermal characterization data (DSC) of PBCE and P($BCE_xBPripol_y$) films before and after hydrolytic tests at 70°C, together with those of the relative blanks.

Samples	Days	T_g	Δc_p	T_m	ΔH_m	T_m	ΔH_m	T_m	ΔH_m
		°C	J/g·°C	°C	J/g	°C	J/g	°C	J/g
PBCE	0	10	0.055	42	0.3	-	-	166	30
	blank							167	30
	2							166	29
	30							166	34
	45							165	35
	60							162	40
P($BCE_{95}BPripol_5$)	0	-9	0.062	42	0.7	-	-	157	20
	blank							158	26
	2							157	25
	30							158	30
	45							158	31
	60							153	32
P($BCE_{85}BPripol_{15}$)	0	-29	0.160	41	1	-	-	137	19
	blank			47	1.5	80	3.8	137	15
	2			48	1.3	93	3.3	138	17
	30			48	0.5	98	1.1	142	22
	45			47	0.3			138	41
	60			48	0.4			135	43
P($BCE_{75}BPripol_{25}$)	0	-38	0.197	41	1	-	-	116	12
	blank			47	1.4	81	3.7	119	8
	2			47	1.2			94<br>115	25
	30			46	0.8			103<br>116	24
	45			46	0.4			104<br>122	30
	60			43	0.2			107<br>121	37
P($BCE_{65}BPripol_{35}$)	0	-42	0.296	45	3			98	4
	blank			47	1.4			82	10

2	46	2.3	90	13
30	45	1.2	98	15
45	45	1.2	101	15
60	43	0.4	104	14

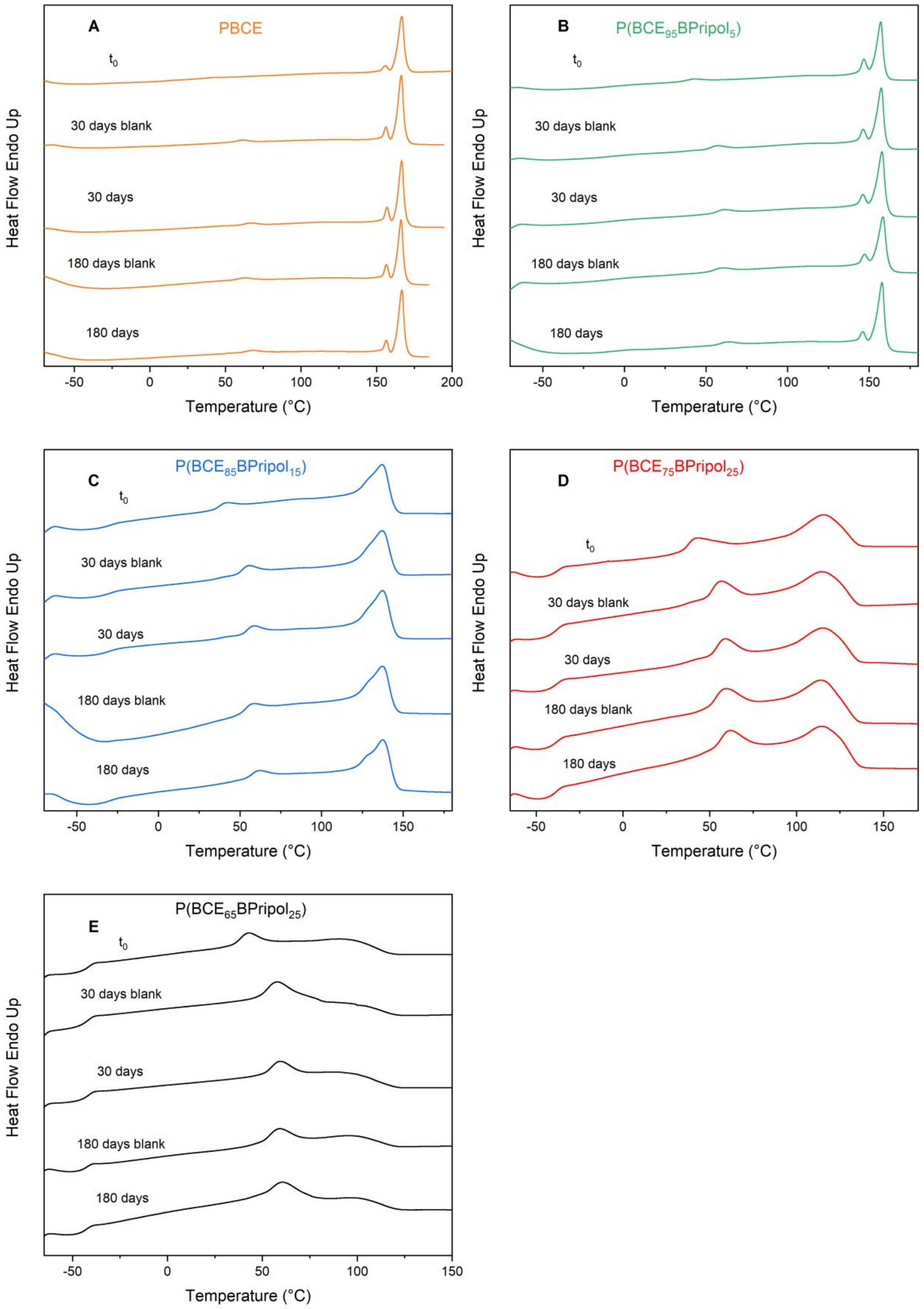

Figure S3. I DSC scans of PBCE and P($BCE_xBPripol_y$) films before and after hydrolytic tests at 37°C, together with those of blanks.

Table S3. Thermal characterization data (DSC) of PBCE and P($BCE_xBPripol_y$) films after hydrolytic tests at 37 °C, together with those of the relative blanks.

Samples	Days	T_g	ΔC_p	T_m	ΔH_m	T_m	ΔH_m
		°C	J/g °C	°C	J/g	°C	J/g
PBCE	0	10	0.055	42	0.3	166	30
	30 blank	n.d.	n.d.			166	29
	30	n.d.	n.d.			167	28
	180 blank	n.d.	n.d.			166	27
	180	n.d.	n.d.			167	27
P($BCE_{95}BPripol_5$)	0	-9	0.062	42	0.7	157	20
	30 blank	n.d.	n.d.			157	22
	30	n.d.	n.d.			158	24
	180 blank	n.d.	n.d.			158	22
	180	n.d.	n.d.			158	24
P($BCE_{85}BPripol_{15}$)	0	-29	0.160	41	1	137	19
	30 blank	n.d.	n.d.	55	2	137	17
	30	n.d.	n.d.	58	1	138	18
	180 blank	n.d.	n.d.	57	2	138	17
	180	n.d.	n.d.	61	1	138	18
P($BCE_{75}BPripol_{25}$)	0	-38	0.197	41	1	116	12
	30 blank	-39	0.2	56	4	116	12
	30	-39	0.221	59	3	116	12
	180 blank	-38	0.225	59	4	115	13
	180	-38	0.234	61	3	117	13
P($BCE_{65}BPripol_{35}$)	0	-42	0.296	45	3	98	4
	30 blank	-43	0.464	58	7	104	2
	30	-43	0.195	59	5	99	2
	180 blank	-42	0.234	59	5	101	3
	180	-43	0.299	60	5	102	3

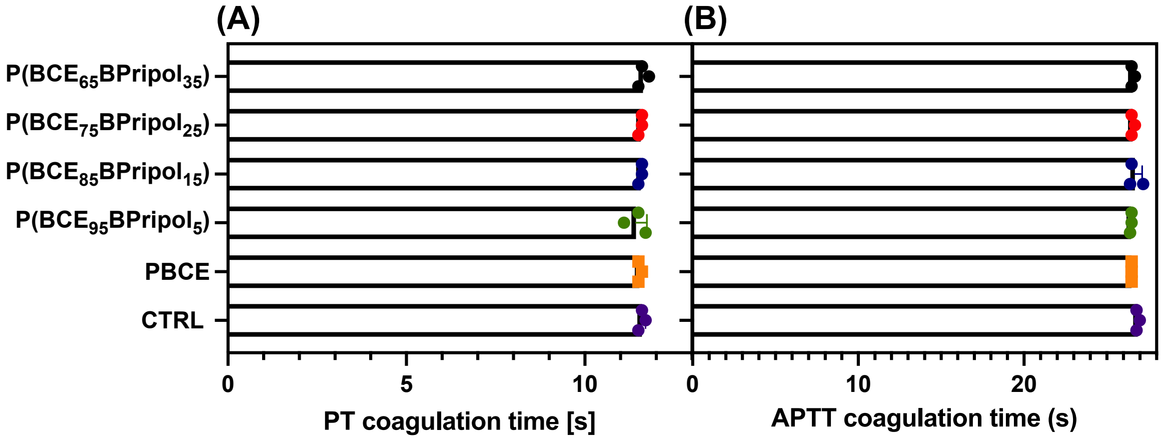

Figure S4. PT (**A**) and APTT (**B**) values of the platelet poor plasma (PPP) in contact for 1 h at 37 ˚C with the different films. CTRL represents PPP incubated in test tubes with no material present. Results are expressed as seconds (s) and presented as mean ± SD (N = 3)

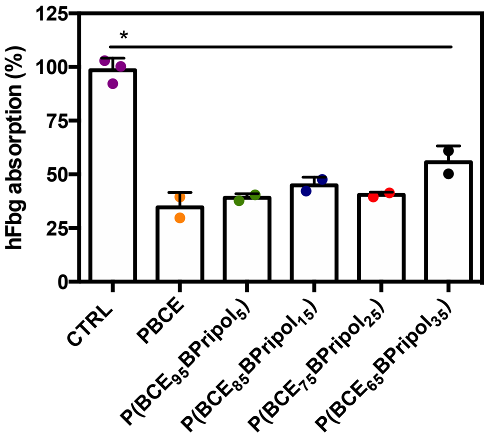

Figure S5. Absorption of a solution of human fibrinogen (hFbg) absorption on the different films and assessed through an anti-fibrinogen HRP-conjugated antibody and quantified via ELISA assay. Data are expressed as percentage related to hFbg added (10 ug/mL) on each sample set as 100% and presented as mean ± SD (N = 3). CTRL represents hFbg absorption on high binding well plates. Symbol (*) indicate statistical significance vs. CTRL (*: $p < 0.05$).
